# Computational identification of putative lincRNAs in mouse embryonic stem cell

**DOI:** 10.1038/srep34892

**Published:** 2016-10-07

**Authors:** Hui Liu, Jie Lyu, Hongbo Liu, Yang Gao, Jing Guo, Hongjuan He, Zhengbin Han, Yan Zhang, Qiong Wu

**Affiliations:** 1 School of Life Science and Technology, State Key Laboratory of Urban Water Resource and Environment, Harbin Institute of Technology, Harbin 150001, China; 2 Dan L. Duncan Cancer Center, Department of Molecular and Cellular Biology, Baylor College of Medicine, Houston, Texas, 77030, USA; 3 College of Bioinformatics Science and Technology, Harbin Medical University, Harbin, 150081, China

## Abstract

As the regulatory factors, lncRNAs play critical roles in embryonic stem cells. And lincRNAs are most widely studied lncRNAs, however, there might still might exist a large member of uncovered lncRNAs. In this study, we constructed the de novo assembly of transcriptome to detect 6,701 putative long intergenic non-coding transcripts (lincRNAs) expressed in mouse embryonic stem cells (ESCs), which might be incomplete with the lack coverage of 5′ ends assessed by CAGE peaks. Comparing the TSS proximal regions between the known lincRNAs and their closet protein coding transcripts, our results revealed that the lincRNA TSS proximal regions are associated with the characteristic genomic and epigenetic features. Subsequently, 1,293 lincRNAs were corrected at their 5′ ends using the putative lincRNA TSS regions predicted by the TSS proximal region prediction model based on genomic and epigenetic features. Finally, 43 putative lincRNAs were annotated by Gene Ontology terms. In conclusion, this work provides a novel catalog of mouse ESCs-expressed lincRNAs with the relatively complete transcript length, which might be useful for the investigation of transcriptional and post-transcriptional regulation of lincRNA in mouse ESCs and even mammalian development.

The mouse is the acknowledged model organism and widely used in studies of the mammalian development and human disease^1^,^2^,^3^. Embryonic stem cells (ESCs) are pluripotent stem cells derived from the preimplantation embryo, which could be able to self-renew and to generate differentiated functional cell types^4^. Due to their developmental potential in cell biology, embryonic stem cells are widely studied in both basic and biomedical researches, as a model system to investigate transcriptional regulatory role in early development^5^. Long noncoding RNAs (lncRNAs) are defined as the RNA transcripts with the length longer than 200 nucleotides and no open reading frame (ORF)^6^. And the intergenic transcripts are most widely studied lncRNAs. Comparing with coding RNAs, lncRNAs often show lower expression abundance and evolutionary conservation^7^. lncRNAs share many genomic features with coding RNAs, for instance, most of which are Pol II transcripts with a poly-A tail and 5′ capping, and also have exons and introns^6^. LncRNAs are crucial regulatory factors in many biological processes including gene silencing^8^, imprinting^9^, and development^7^,^10^.

In recent years, the roles of lncRNAs in ESCs attract more and more attention. And abundant researches showed that lncRNAs play critical regulatory roles in ESCs^5^,^11^,^12^,^13^,^14^. Guttman *et al*. performed loss-of-function studies on long intergenic non-coding transcripts (lincRNA) in mouse embryonic stem cells (mESCs), and the results showed that the lincRNAs are involved in the gene expression regulatory networks and embryonic stem (ES) cell differentiation^12^. As the important regulatory factors in embryonic stem cells and other cells, tissues and organs of organism, lncRNAs perform critical functions in development and disease. The mammalian genome encodes many thousands of lncRNAs, and currently, more than 6,000 lncRNAs, including over 2,500 lincRNAs in the mouse genome which were annotated by experimental validation in the GENCODE project^15^. There might still exist a large member of uncovered lncRNAs scattered in the genome. Similar to many computational methods and platforms developed for miRNAs^16^,^17^, many computational efforts have been paid for lncRNAs. As the development of the relevant biotechnologies, RNA-Seq has become the comprehensive way to detect the novel lncRNAs and profile the lncRNA expression^18^,^19^,^20^,^21^. Lv *et al*. identified the long non-coding RNAs over mouse brain development based on the RNA-Seq data, chromatin and genomic features by machine learning method^22^. A set of lincRNAs was detected by Guttman *et al*. by RNA-Seq data in three moue cell types, including embryonic stem cells (ESCs), neural progenitor cells (NPCs) and mouse lung fibroblasts (MLFs)^23^. In the study of the Ramos *et al*., thousands of lncRNAs of adult neural stem cells were identified from *de novo* reconstructed transcriptome sourced from the Illumina RNA-Seq^24^. Lv *et al*. also used the transcriptomic data to identify and characterize the long non-coding RNAs of mouse embryonic brain development^21^.

De novo lncRNA identification from RNA-Seq might result in the acquisition of the partial transcripts. Generally, Lower expression abundance of lncRNAs compared with protein coding transcripts may lead to partially annotated lncRNA transcripts in known lncRNA sets^25^. For the sample preparation in RNA-Seq, the RNA degradation from 5′ end might lead to biases along the transcript with the 5′ end under representation^26^. GC rich regions such as the CpG islands (CGIs) may also lead to the sequencing bias, and especially the low depth of coverage, because high C+G content regions are prone to resist the DNA denaturation during amplification^26^,^27^. However, the RNA fragmentations based on RNA-Seq are often depleted for transcript ends^28^. The polyA+ RNA-Seq experiments by first-strand synthesis primed with oligo-dT accurately annotate the 3′ ends but often fail to cover the 5′ ends^28^, resulting in the biased enrichment of the 3′ ends relative to 5′ ends^29^. And most Illumina RNA-Seq datasets have shown the 3′ end coverage enrichment^30^. Thus, it is difficult to discover and annotate the complete lncRNA transcripts with the 5′ end absence based on RNA-Seq, which would be the obstacle to determine the precise transcript boundaries and quantify the transcript expression^18^. Also, the partial transcripts may lead to misleading results of the subsequent functional annotation of the lncRNAs^18^. Conventional 5′-RACE experiment is the best method to capture the 5′ complete cRNAs. Other sequencing technologies to characterize the site of 5′ transcript termini include CAGE (cap-analysis of gene expression), nanoCAGE^31^, PET (paired-end tag sequencing)^32^, RAMPAGE (RNA Annotation and Mapping of Promoters for the Analysis of Gene Expression) methods^33^,^34^. CAGE is often used as the complementary technology of RNA-Seq, helps improve the incomplete transcripts^35^.

In this study, we collected paired-end RNA-Seq data of mouse ESCs and other tissues with long reads derived from the public source to construct the *de novo* assembly which could facilitate the detection of the uncovered lincRNA transcripts in the genome. Next, the transcripts that expressed in the mouse ESCs were collected as the novel lincRNA transcripts in the mouse ESCs. Because of the potential biases caused by the RNA-Seq technology including the sample preparation and other reasons, the completeness of the transcripts was estimated by CAGE data from the Fantom5 project^35^. A significant proportion of the novel lincRNA transcripts might be incomplete with the obvious 3′ end bias. To overcome the 3′ end bias and acquiring the comparative full-length transcripts, we combined the sequence features and the epigenetic modifications to construct a prediction model of lincRNA TSS (Transcription Start Site) proximal regions based on the machine learning method RBF SVM. We proved the robustness of the prediction model by the 10-fold cross-validation and the independent test data set. And using this model, more than 1,000 novel transcripts were corrected. Finally, a set of relatively complete lincRNA transcripts expressed in the mouse embryonic stem cells (ESCs) was gained, which might be useful for the investigation of the transcriptional regulation and the posttranscriptional regulation of lincRNA in mouse ESCs and even the mammalian development.

## Results

### Identification of novel lincRNA transcripts in mouse ESCs

Mouse Embryonic stem cells (ESCs) play the important roles in mammalian early development. To systematically identify putative lincRNA with potential roles in mouse ESCs, 14 RNA-Seq data were collected in ESCs. Due to the lower expression level of lincRNAs compared to that of protein coding RNAs, RNA-Seq data from other cells and tissues in the mouse embryonic development were added to guide ESC transcriptome construction (Supplementary Table S1). Considering the effects of read length and sequencing depth of RNA-Seq on the identification of transcriptional isoforms, the collected RNA-Seq data were restricted to paired-end sequencing data with the read length longer than 50 bp.

**Figure 1:**
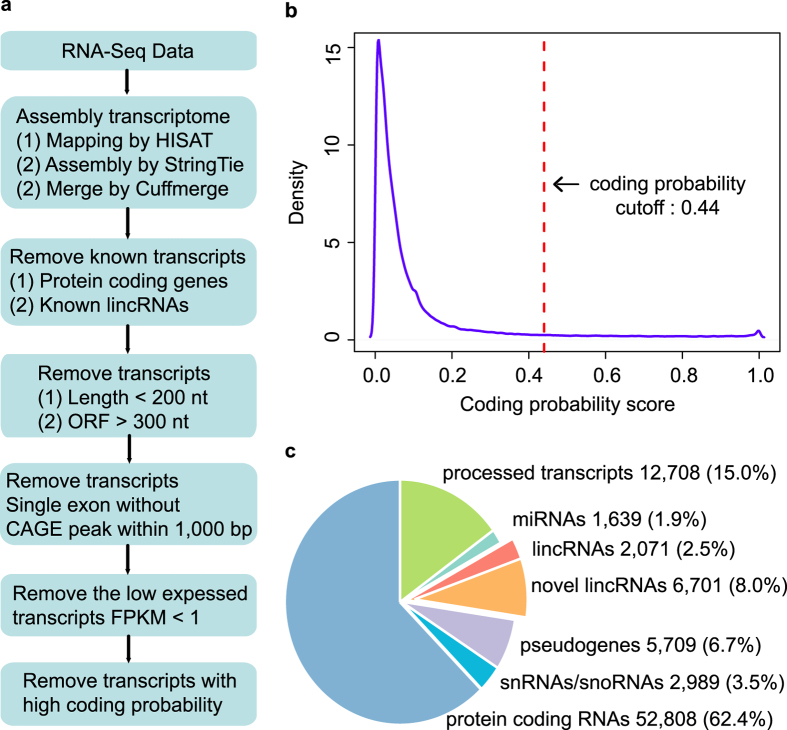
The identification of putative lincRNAs expressed in mouse ESCs. (**a**) Overview of the identification pipeline for novel lincNRAs expressed in mouse ESCs. (**b**) The distribution of the coding probability for putative lincRNAs by CPAT. (**c**) Pie chart of composition of quantities of putative lincRNA, known lincRNA and other transcripts in the mouse ESC assembly.

Following the pipeline of the novel lincRNA identification (Fig. 1a), we obtained a total of 446,488 transcripts after merging the RNA-Seq transcriptome with GENCODE^15^ mouse annotation (see Methods). After removing the transcripts overlapped with known annotations, we only kept 188,386 transcripts as the candidate lincRNA set. As a result, we obtained 159,871 transcripts with length > 200 nt and ORF < 300 nt^22^. For the single exon transcripts, only the transcripts overlapped with the Fantom5 CAGE peaks within 1,000 bp of the 5′ end regions were retained. The lowly expressed transcripts with FPKM value <1 in the mouse ESCs were removed. Finally, a set of 6,701 transcripts were obtained as the novel putative lincRNAs in mouse ESCs, when filtering out the transcripts with high coding potential (threshold of 0.44 by CPAT^36^) (Fig. 1b).

The novel putative lincRNAs were compared with the known transcripts from Ensembl transcript category. It is shown for the reconstructed transcriptome that, protein coding transcripts and processed transcripts accounting for 62.4% and 15.0%, respectively (Fig. 1c). While the small non-coding RNAs such as miRNAs and snRNAs/snoRNAs only accounted for 1.9% and 3.5%, respectively (Fig. 1c). And the novel lincRNAs also accounted for 8% of the transcriptome, but much higher than known lincRNAs (2.5%) (Fig. 1c).

### Incompleteness of Novel lincRNA transcripts

**Figure 2:**
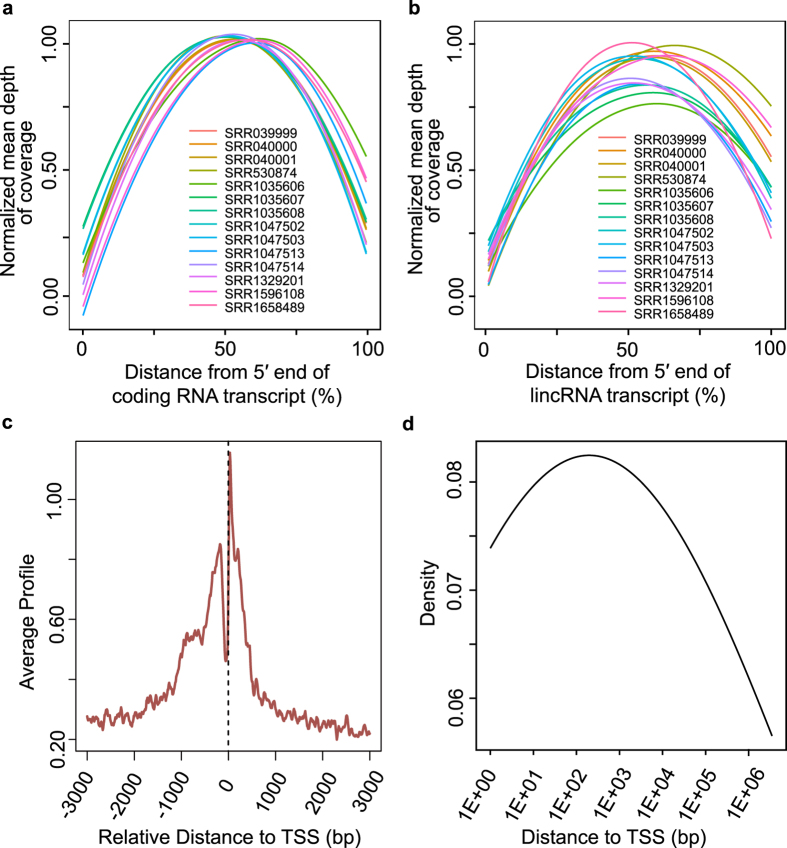
Estimation of the transcript completeness. (**a**) Coverage depth of coding transcripts. The read coverage of 14 RNA-Seq data for known protein coding transcripts. (**b**) Coverage depth of lincRNAs. The read coverage of 14 RNA-Seq data for known lincRNA transcripts. (**c**) The average profile of CAGE tags around the TSS regions of putative lincRNAs. (**d**) The distribution of distance between CAGE peaks and TSS of putative lincRNAs.

The inherent problems of RNA-Seq result in the detection of partial transcripts with the lack coverage of 5′ ends^26^,^27^,^29^. To systematically and comprehensively estimate the completeness of transcripts, the expression levels and the coverage depth of the aligned reads of known protein coding transcripts and lincRNA transcripts were examined. 259 lincRNA transcripts and 18,585 protein coding transcripts were obtained with the expression value ≥0.5 FPKM in mouse ESCs. The lengths of all the expressed protein coding and lincRNA transcripts were normalized as 100 nt. The normalized read coverage was shown for known lincRNA and protein coding transcripts in Fig. 2a,b, from which we found 3′ bias of the protein coding transcripts, with the mean coverage of 3′ ends was higher than that of 5′ ends. As expected, the 3′ end bias is more pronounced for known lincRNA transcripts. For instance, a known protein coding transcript, ENSMUST00000042734 with 2 exons located in chr1 forward strand and length of 3,008 bp is nearly depleted of RNA-Seq signal in the first exon, as is seen in the aligned read coverage signals from all mouse ESCs RNA-Seq data (Supplementary Fig. 1). The situation is also available in another protein coding transcript ENSMUSG00000035462 and two multi-exon lincRNA transcripts ENSMUST00000133808 and ENSMUST00000153581 with the lower aligned reads covered in 5′ end (Supplementary Figs 2, 3 and 4). Thus, the depth of the sequencing aligned reads of RNA-Seq may be insufficient to identify the 5′ ends of putative lincRNAs^28^, though the sequencing depth is relatively high.

To investigate whether the 5′ bias could be solved, the robust CAGE peaks from Fantom5 were used to estimate the incompleteness of the novel lincRNA transcripts^35^,^37^, which is the biotechnology capturing the TSS and the region of its 30 bp downstream. The average profile of CAGE tags in TSS proximal regions of novel putative lincRNAs in mouse ESCs was shown in Fig. 2c. We found the bimodal distribution and a peak in the upstream region of the meta lincRNA TSS regions. Further, a large proportion of distances between lincRNA 5′ ends and CAGE peaks were in the range from 100 bp to 1,000 bp, which may be accounted for by the lack of 5′ ends of the lincRNA transcripts detected by RNA-Seq data and can be used to correct the 5′ ends of the novel putative lincRNAs (Fig. 2d).

### The Comprehensive genomic and epigenetic Analysis of lincRNA TSS proximal region

To correct the 5′ ends of the putative lincRNAs, we primarily investigated the transcriptional regulation patterns of the lincRNA TSS proximal regions. Due to the well-understanding of transcriptional regulation of protein coding genes, the TSS proximal regions of the 259 expressed lincRNAs and 233 expressed protein coding genes closest to these lincRNAs in mouse ESCs (FPKM ≥ 0.5) were compared.

**Figure 3:**
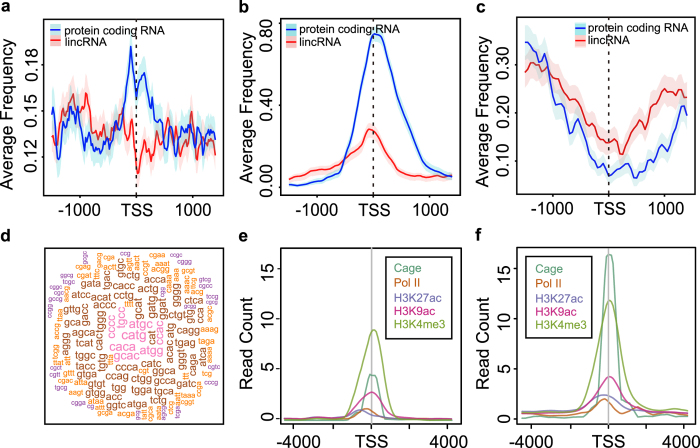
The comparison of sequence and epigenetic features between known lincRNAs and their neighbour protein coding transcripts. (**a**) The palindrome distribution in the TSS proximal regions of known lincRNAs and their neighbouring protein coding transcripts. (**b**) The CGI coverage frequency in the TSS proximal regions of known lincRNAs and their neighbouring protein coding transcripts. (**c**) The repeat element frequency in the TSS proximal regions of known lincRNAs and their neighbouring protein coding transcripts, except for simple repeats, low complexity regions and satellite repeats. Shadow regions in (**a–c**) represent the 5–95% bootstrap confidence intervals of the statistics. (**d**) The over-represented and under-represented k-mers of known lincRNAs and their neighbouring protein coding transcripts. (**e**) The distribution of chromatin modifications in TSS proximal regions of known lincRNAs. (**f**) The distribution of chromatin modifications in TSS proximal regions of the protein coding transcripts closest to the known lincRNAs.

The genomic sequence features were compared between the TSS proximal regions of lincRNAs and protein coding genes. Palindromes (palindromic sequences) usually locate at the promoter regions of protein coding RNAs and are often identified as the putative binding sites as regulatory elements. CGI is the well-known cis-regulatory element overlapping with the promoter regions of many genes in mammalian genomes. Repeat elements are widespread in the mammalian genomes. And the frequency distributions of the three sequence features palindromes, CGI and repeat elements were investigated by calculating the coverage ratio in the sliding window of 300 bp with a step of 50 bp in the promoter regions of protein coding genes and lincRNAs. Palindromes frequency of TSS regions of lincRNA and protein coding genes were compared and shown in Fig. 3. The two sequence features were significantly differential in the TSS proximal regions between the lincRNAs and protein coding genes^38^. The palindromes frequency around protein coding RNAs was a peak, but on the contrary, there was a trough around the lincRNA TSS proximal regions (Fig. 3a). Further, the coverage of CGI and repeat elements in the two TSS proximal region sets were also compared (except for simple repeats, low complexity regions and satellite repeats)^38^ (Fig. 3b,c). Though both two TSS proximal region sets lack repeat elements, the CGI coverage in TSS regions of protein coding RNAs was much higher than that of lincRNAs, and the repeat element coverage of protein coding RNAs was lower than that of lincRNAs. To get the over-represented and under-represented DNA words, the Obs/Exp ratios of K mer (K = 2, 3, 4) for these two gene sets were calculated, respectively. And then, the Obs/Exp ratios were compared by calculating the fold change, in which the DNA words with the odd ratios >1.2 or <0.8 were retained. 61 DNA words were obtained and considered as the specific base alignments between lincRNA and protein coding RNAs (Fig. 3d). All these results indicated that the TSS proximal regions of lincRNAs had specific genomic features which were significant different from protein coding RNAs.

Furthermore, we investigated the histone modifications especially active regulatory elements in the two TSS proximal region sets, respectively. In Fig. 3e,f, the histone modifications of the two TSS proximal region sets were enriched in the lincRNA TSS proximal regions with the intensity of all the three modifications much lower than those in protein coding TSS proximal regions. CAGE and PolII also show similar patterns (Fig. 3e,f). Taken together, the epigenetic features of the lincRNA TSS proximal regions could be used as the basis of the 5′ end correction in the subsequence analysis.

### Feature selection for lincRNA TSS region identification

The GENODE annotation combines the Havana manual gene annotation and the Ensembl automated gene annotation based on the experimental evidence. So the 259 GENCODE lincRNAs expressed in mouse ESCs were considered as the golden standard lincRNA set, and the regions from −1 kb to +0.5 kb with respect to TSS of these lincRNAs were defined as the TSS proximal regions (training positive set). The TSS regions were compared with the random regions (training negative set) from unannotated regions with the size ranging from −1 kb to +0.5 kb within the regions of ±10 kb relative to the lincRNAs TSS sites.

**Figure 4:**
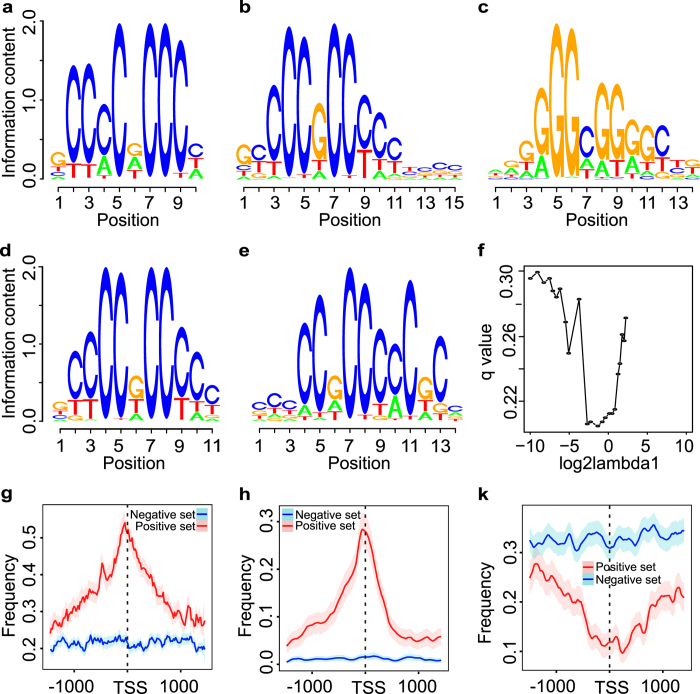
Sequence feature selection for prediction model. (**a–e**) show five representative motifs of the prediction model, including KLF5, SP2, GC-box, SP1 and EGR1. (**f**) SCAD feature selection. K-mers selection with the best performance to distinguish the lincRNA TSS regions from the random regions. (**g–k**) show the sequence features between the positive and negative sets, including CpG o/e (**g**) CGI coverage (**h**) and repeat element (without simple repeats, low complexity regions and satellite repeats) coverage (**k**). The red line represents the positive set, and the blue line represents the negative set. Shadow region represents the 5–95% bootstrap confidence intervals of the statistics.

The sequences of lincRNA proximal TSS and random regions were scanned for the occurrence of 606 JASPER CORE TF motifs^39^ by FIMO which is an analysis tool of MEME^40^. And the enriched or depleted TF motifs in the lincRNA TSS proximal regions were inquired, which were compared with random regions. Finally, five TF motifs: KLF5, SP2, GC-box, SP1 and EGR1 were identified as the significant discriminative sequence features between lincRNA TSS proximal and random regions by Fisher exact test (FDR < 0.01) (Fig. 4a–e). To find the representative substrings for lincRNA TSS proximal regions, k-mer analysis was performed. We only estimated the probability of the combinations of the four bases (A, T, C, and G) when k was equal to 2, 3 and 4. Through the analysis of the unbiased penalty method SCAD (smoothly clipped absolute deviation)^41^,^42^, only four features (CA, CG, AAT and ACA) from the 336 k-mer features could distinguish the lincRNA TSS proximal regions from the random regions with the least error rate (Fig. 4f).

The specific genomic and chromatin features of lincRNA TSS proximal regions which were significantly differential from protein coding TSS proximal regions were obtained and compared with the random regions. It was revealed that the CpG o/e ratio and CGI coverage features were quite different in the TSS regions between lincRNAs and random regions (Fig. 4g,h). Repeat elements frequency (without simple repeats, low complexity regions and satellite repeats) also distinguished the lincRNA TSS proximal regions from random regions (Fig. 4k). The active chromatin modifications of lincRNA proximal TSS regions were also significantly different from random regions, including PolII, CAGE and three histone modifications (H3k4me3, H4k9ac and H3k27ac) (Supplementary Fig. 5). These characteristic sequence and chromatin features were candidates for the subsequence lincRNA TSS proximal region identification based on machine leaning method.

### Prediction of lincRNA TSS proximal region through sequence contexts and chromatin modifications

Machine learning methods are widely used to solve the biological problems, such as HMM^43^, random forest^44^, SVOR^45^ and so on. In this study, to construct a robust and precise prediction model, the different feature types were analyzed by RBF SVM (Support Vector Machine). As described above, the TSS proximal regions of the 259 GENCODE lincRNAs which expressed in mouse ESCs were selected as the positive training set, and the random unannotated regions with respect to the positive TSSs were defined as the negative set.

**Figure 5:**
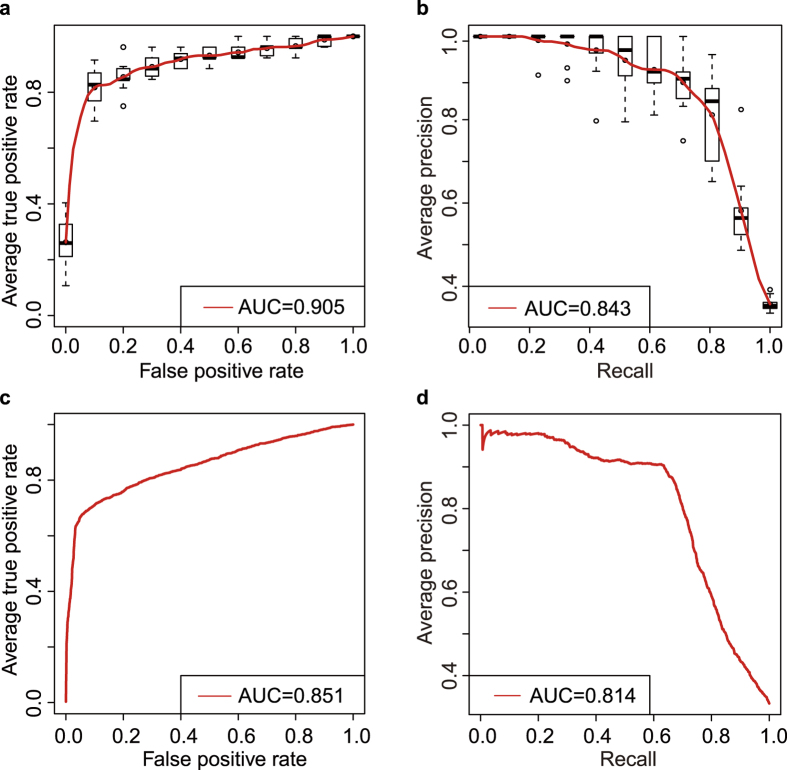
Performance evaluation of the predict model. (**a**) The average ROC curve (Receiver Operating Characteristic curve) for 10-fold cross validation of the predict model. (**b**) The average PRC curve (Precision-Recall curve) for 10-fold cross validation of the predict model. Boxplots around the average curves in (**a,b**) indicate the variations. (**c,d**) show the ROC and PRC curves for the testing set of the predict model, respectively. AUC in (**a–d**) represents the Area Under Curve.

Initially, models were constructed by feature groups of different categories. The feature CAGE was firstly assessed by SVM, because of its excellent capacity in identifying the transcriptional start sites (TSS) and monitoring the dynamics for transcription TSSs for different tissues or cells. For SVM classifier with CAGE alone, the accuracy was only 0.74 for the 10-fold cross-validation with AUC under the ROC curve (AUC) equals to 0.79 (Table 1). For the classifiers with the k-mer, CGI and CpG o/e features, the accuracies were 0.79 and 0.80, respectively, comparable to CAGE, and also AUCs under the ROC curve (0.76 and 0.75, respectively). And the classifier with repeat element (without simple repeats, low complexity regions and satellite repeats) only achieves the lowest accuracy for model evaluation (AUC = 0.63). Next, the classifier with the representative motifs (KLF5, SP2, GC-box, SP1 and EGR1) was also assessed. The motif classifier achieved a higher accuracy and AUC under the ROC curve (0.82), indicating that the five representative motifs are better predictor of lincRNA TSS regions than other sequence features and CAGE. Finally, the chromatin modifications (Pol II, H3k4me3, H4k9ac and H3k27ac) achieved the highest AUC (0.85), although the accuracy was lower than that of the classifier trained by the five representative motifs, suggesting that chromatin modifications may have more regulatory ability than TFs. After combining all features, including the sequence contexts and the chromatin modifications, we found the classifier achieved the best performance with the predictive accuracy and AUC equals to 0.88 and 0.90, respectively, in 10-fold cross-validation (Table 1 and Fig. 5a,b), indicating that the combined classifier of the sequence contexts and the chromatin modifications may be the best model for lincRNA TSS proximal region prediction.

An independent lincRNA TSS proximal region set was needed to assess the robustness of the combined classifier with the measures. Due to lack of the reference lincRNA set, and because of the reliability of the GENCODE annotation, 2,254 GENCODE lincRNAs were used as the testing set, expect for the 259 lincRNAs used as the training set. And the TSS proximal regions with the range from −1 kb to +0.5 kb with respect to the corresponding TSS were used to assess the combined classifier. The predictive accuracy was 0.85 and the AUC under the ROC curve equals to 0.85 for the testing set (Table 1 and Fig. 5c). The AUC under PRC curve of the testing set equals to 0.81 (Fig. 5d), indicating that the combined classifier of the sequence contexts and chromatin modifications was a robust and precise predictive model for lincRNA TSS proximal regions.

For the genome-wide unannotated intervals, the regions for prediction were obtained by the sliding window method with the window size of 1,500 bp and the step of 300 bp. And these intervals with the equal length were assessed by the combined classifier. Finally, 108,211 intervals were identified as the putative lincRNA TSS proximal regions and were used for the subsequent analysis.

### Correction of novel lincRNA TSS proximal regions

As mentioned above, ~0.11 million potential lincRNA TSS proximal regions were identified, which were used to correct the putative lincRNA 5′ ends in the mouse ESCs. For each putative lincRNA, in the upstream 1,000 bp region of the TSS, the closest potential lincRNA TSS proximal region was considered as the new TSS region. The site locating in 1,000 bp of this region with the length 1,500 bp was defined as the new putative TSS for the corresponding lincRNA. Finally, among the 6,701 putative lincRNAs, the 5′ ends of 1,293 lincRNAs were corrected (Supplementary Table S3).

**Figure 6:**
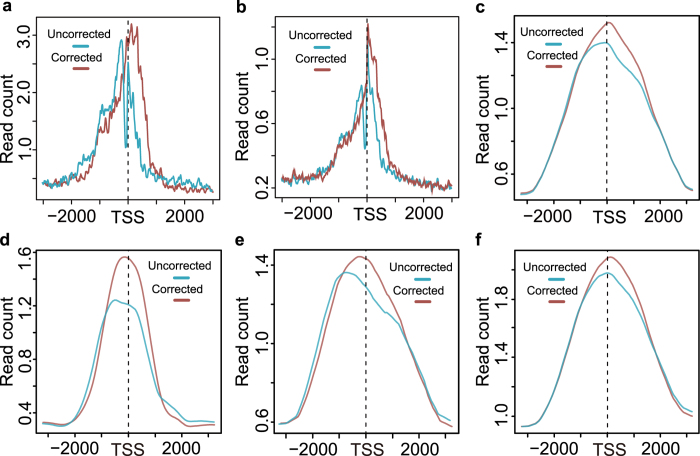
The performance estimation of the lincRNA 5′ end correction. (**a**) The average profile of CAGE tags around the TSS regions of 1293 putative lincRNAs before and after correction. (**b**) The average profile of CAGE tags around the TSS regions of 6701 putative lincRNAs before and after correction. (**c–f**) The average profiles of DHS tags (**c**) H3k4me3 tags (**d**) H3k9ac (**e**) and H3k27ac (**f**) around the TSS regions of 6701 putative lincRNAs before and after correction. Red line corresponds to lincRNA TSS proximal regions before correction, and blue line corresponds to lincRNA TSS proximal regions after correction in (**a–f**).

To estimate the performance of the lincRNA 5′ end correction, the TSS proximal regions before and after correction were characterized. Firstly, the TSS proximal regions of 1,293 corrected lincRNAs before and after correcting were compared. It was shown from the average profiles of CAGE tags (Fig. 6a) that, the uncorrected average profile had two peaks near TSS and its upstream region, indicating that these lincRNAs might be the incomplete transcripts with the lack of 5′ ends. And after corrected, it was shown that the average profile had only one peak around TSS, which meant that CAGE peaks were located around TSS for the majority of the 1,293 lincRNAs (Fig. 6a). The meta-transcript profiles also confirmed that the transcripts tended to be more complete after correcting the 5′ ends (Supplementary Fig. 6). And next, all the putative lincRNAs expressed in mouse ESCs were assessed by CAGE tags (Supplementary Table S4), which was shown in Fig. 6b. The average CAGE tag profile had only one peak around TSS after correcting compared to the uncorrected profile with two CAGE peaks which had one peak locating TSS upstream (Fig. 6b). Taken together, these results demonstrated the usefulness of the lincRNA 5′ end correction.

The TSS proximal regions of the putative lincRNAs expressed in mouse ESCs were characterized through DNaseI hypersensitive sites identified by DNase-Seq which had better sensitivity than FAIRE-Seq at promoter regions^46^. It was shown that the corrected lincRNAs of the 6,701 lincRNAs had more enriched hypersensitive sites signal intensity around TSS compared to that of uncorrected lincRNAs (Fig. 6c). And the lincRNAs before and after correction were also characterized by the histone modifications, including H3k4me3, H3k9ac and H3k27ac (Fig. 6d–f), which were known active regulatory elements associated with promoter^47^. As expected, the signal intensities of the three active histone modifications were more enriched around TSSs of the lincRNAs with the 5′ ends corrected. In summary, we demonstrated that, the putative lincRNA transcripts expressed in mouse ESCs tended to be relatively complete after the correction of the 5′ ends.

### Function annotation of the putative lincRNAs expressed in mouse ESCs

Non-coding RNA could play important roles in the regulation of gene expression at transcriptional, epigenetic and post-transcriptional levels in mammalian development. Extensive researches predict the functions of genes or identify the disease-related genes based on computational strategies^48^, especial for miRNA^49^,^50^. Unlike miRNA, the functions of only dozens of lincRNAs appear in these years. In this study, guilt-by-association method was used to predict the functions of the putative lincRNAs. For the 6,701 putative lincRNAs expressed in mouse ESCs, a subset of 527 lincRNAs with expression FPKM value ≥0.5 were obtained. And 11,810 coding genes with expression FPKM value higher than 0.5 in mouse ESC samples were also collected. The association of each putative lincRNA with the GO terms was assessed by the correlations between putative lincRNAs and the protein coding genes through Kolmogorov-Smirnov test using GSEA^51^,^52^.

**Figure 7:**
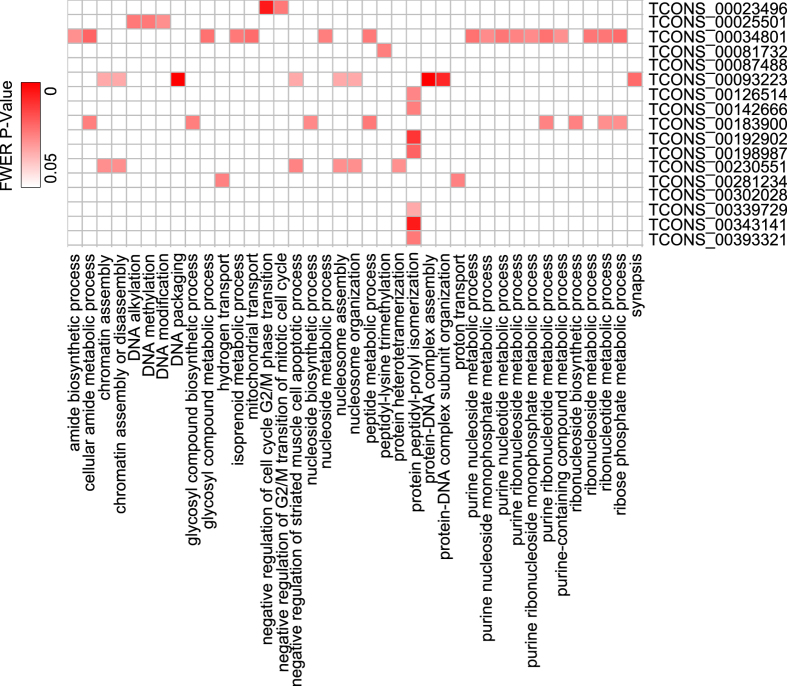
The GO biological processes of the putative lincRNAs annotated through guilt-by association method.

Finally, among the 527 selected putative lincRNAs, only 43 lincRNAs were annotated in the three GO categories, including Biological Process (BP), Cellular Component (CC) and Molecular Function (MF). For the 14,282 GO BP terms, only 39 terms were significantly associated with 17 putative lincRNAs, including the biological processes of chromatin assembly or disassembly, DNA methylation, DNA modification, protein peptidyl-prolyl isomerization and so on (Fig. 7 and Supplementary Table S5). And the GO BP term, protein peptidyl-prolyl isomerization, was mapped to 7 lincRNAs, which was the GO term mapped to the most lincRNAs. The putative lincRNA, TCONS_00034801, was annotated with 17 GO BP terms, indicating that this lincRNA might play crucial functions in ESCs and even in embryonic development stage of mouse. The putative lincRNAs were also mapped with GO CC and MF terms. And only 23 GO terms of 4,842 GO CC terms were associated with 24 putative lincRNAs, including the important cellular components, including DNA-directed RNA polymerase II, core complex, DNA packaging complex, protein-DNA complex and so on (Supplementary Fig. 7 and Supplementary Table S6). Finally, among the 527 putative lincRNAs, 19 lincRNAs were annotated with 16 terms of 1,700 GO MF terms. The GO MF term, peptidase regulator activity, could be associated with the most putative lincRNAs (TCONS_00354155, TCONS_00215988, TCONS_00176787 and TCONS_00119949) (Supplementary Fig. 8 and Supplementary Table S7). Consistent with prior studies^5^,^12^,^14^, these results indicated that the putative lincRNAs might play the important roles in epigenetic regulation, transcriptional regulation and posttranscriptional regulation of mouse ESCs.

## Discussion

In this study, we constructed the *de novo* assembly to detect 6,701 putative lincRNAs expressed in the mouse embryonic stem cells (ESCs) by collecting multiple RNA-Seq data of mouse ESCs. When investigating the sequencing read coverage depth of known lincRNAs and protein coding transcripts, it was revealed that there was insufficient read coverage in the regions of the 5′ ends for both lincRNA and protein coding transcripts. The novel lincRNAs expressed in mouse ESCs were assessed by CAGE data, and the result indicated that these transcripts might be incomplete with the insufficient coverage of 5′ends. To characterize the lincRNA TSS proximal regions, the sequence context and epigenetic features of the TSS proximal regions between the known lincRNAs and their closet protein coding transcripts were compared. Next, the prediction model based on RBF SVM was constructed to identify the lincRNA TSS proximal regions on the genome-wide scale by the combination of genome features and the epigenetic modifications. Subsequently, 1,293 lincRNAs were corrected in their 5′ ends using the predicted TSS regions by the SVM model. Finally, 43 putative lincRNAs expressed in mouse ESCs which might play the crucial roles in mouse ESCs were annotated by GO categories through the guilt-by-association GSEA method.

To improve the precision of the novel lincRNA identification, we only use the paired-end RNA-Seq data with the read length longer than 50 bp (Supplement Table 1). When we quantify the expression level of the known lincRNAs in mESCs, it was found that only 259 lincRNAs were associated with the FPKM expression level ≥0.5, which might be caused by the temporal and spatial specificity of lincRNA^53^. And the novel transcripts might be discovered partially due to the low coverage caused by the low expression abundance and the tissue specificity of lincRNA^26^. Therefore, we constructed a comprehensive non-redundant transcriptome assembly from RNA-Seq data sourced from the public repository, including the mouse early embryos and other tissues in the different development stages (Supplement Table 1). For the identification of the novel lincRNAs in mouse ESCs, only the novel assembled isoforms with FPKM ≥1 were considered as the putative lincRNAs in mouse ESCs.

Due to the problems of low expression abundance of lincRNA, RNA library preparation for sequencing, sample preparation and other factors might affect the detection of full-length lincRNA transcripts^14^,^27^. Some of RNA-Seq data were polyA+ RNA by the first-strand synthesis primed with oligo-dT which could annotate 3′ ends more accurately than the 5′ ends. When preparing the sample for sequencing, the RNA degraded from the 5′ ends, which might result in the lack coverage of 5′ ends for the expressed transcripts. And CG-rich could lead to the low read coverage by resisting the DNA denaturation when amplifying^32^. These problems above could result in the identification of the partial transcripts based on RNA-Seq data. So, we investigated the RNA-Seq read coverage depth of the known lincRNA and protein coding transcripts (Fig. 2a,b), and the results showed that both lincRNAs and protein coding transcripts had more read abundance in 3′ ends than in 5′ ends (Fig. 2a,b). We also examined the novel lincRNAs expressed in mouse ESCs using CAGE peaks derived from Fantom5 which could capture the short sequence of 20 to 27 nt corresponding to the transcript TSSs^37^. And in this study, it was revealed that the distances between the putative lincRNAs and the CAGE peaks were around 1,000 bp (Fig. 2d), indicating the incompleteness of the putative lincRNAs. Thus, it was necessary to correct the 5′ ends of the novel lincRNAs expressed in mouse ESCs which were detected by RNA-Seq data.

To address the problem, the lincRNA TSS proximal regions (−1 to +0.5 kb with respect to TSSs) were predicted in the mouse ESCs by the RBF SVM model with the combination of sequence contexts and the chromatin modifications. The prediction model achieved the best performance of 10-fold cross-validation with all features, indicating that it was the robust model by the integration of the sequence contexts and the epigenetic modifications. The model was further tested by an independent data set which was not used in the model training. Due to the lack of the golden standard testing set, this testing set of lincRNAs were the GENCODE lincRNA transcripts with FPKM ≥0.5 in mouse ESCs except for those used in the training set. It was revealed that the prediction model had the capacity of the identification of the lincRNA TSS proximal regions in the genome-wide scale by combining the sequence contexts and the chromatin modifications.

CAGE is one of the most effective methods for the identification of the 5′ transcript termini until recently. As a complementary technology of RNA-Seq, CAGE helps improve the incomplete transcripts^35^. Because of the temporal and spatial specificity of the lncRNA transcripts, the limited cell line or tissue coverage of the TSS set and the small number of the samples might affect the detection of the 5′ end of the lncRNA transcripts by the CAGE and other sequencing technologies, which would be overcome when more CAGE datasets are available.

## Conclusion

In conclusion, this work provides a novel catalog of mouse ESCs-expressed lincRNAs with relatively complete transcript length, which might be helpful for the quantification of the transcript expression and the subsequent functional annotation of the lincRNAs. And the putative lincRNAs expressed in mouse ESCs might be useful for the investigation of the transcriptional regulation and posttranscriptional regulation of the lincRNAs in mouse ESCs and even the mammalian development.

## Methods

### Datasets

The paired-end RNA-Seq data with the read length >50 bp were used to detect novel lincRNAs in mouse ESCs. To precisely identify the putative lincRNAs and avoid the biases caused by the low expression abundance and the high expression specificity, we used the RNA-Seq data derived from ESCs, mouse whole embryos, mouse early embryos and other tissues in the mouse different development stages to guide novel lincRNA assembly (Supplementary Table S1). The mESC RNA-Seq data derived from GSE36026 were used to quantify the expression of known lincRNAs and protein coding transcripts (Supplementary Table S1).

The publicly available mESC ChIP-Seq data of H3k4me3, H3k9ac, H3k27ac and PolII for training prediction model were obtained from ENCODE project with the GEO accession ID GSE31039^54^. For the evaluation of the putative lincRNA TSS proximal regions, the ChIP-Seq data from public database GEO: H3k4me3 (GSE12241), H3k9ac and H3k27ac (GSE40951) were also collected (Supplementary Table S2). The CAGE data were obtained from Fantom5 (functional annotation of the mammalian genome)^37^.

### Publicly available annotations

In this study, the GENCDOE version M2 was obtained as the reference mouse known gene annotation (http://www.gencodegenes.org/mouse_releases/2.html)^15^, and was converted to mm9 assembly version using LiftOver (http://genome.ucsc.edu/cgi-bin/hgLiftOver). The Ensembl^55^ gene annotations version mm9 were also used and collected from UCSC database. The mouse genome sequences (version mm9) were downloaded from the UCSC Genome Browser (http://genome.ucsc.edu). The CGIs and repeat elements annotations for mouse mm9 version were also obtained from UCSC database. The GO term lists of gene sets with the Entrez ID used for the putative lincRNA GO annotation were obtained from Enrichment Map Gene Sets (http://download.baderlab.org/EM_Genesets), including three categories molecular function, cellular component and biological process, which contain 4,842, 1,700 and 14,282 GO terms in the corresponding list, respectively. The file gene2ensembl, which was used for the ID conversion between Entrez ID and Ensembl ID of known lincRNAs and protein coding transcripts, was downloaded from public sources NCBI (ftp://ftp.ncbi.nlm.nih.gov/gene/DATA/).

### Sequencing reads alignment

FastQC was used to examine the quality of the sequencing data. And the sequencing reads passing the quality control were mapped to the mouse reference genome. ChIP-Seq data were aligned to the mouse mm9 genome by Bowtie2 which was the ultrafast and efficient tool for sequencing reads alignments using the default options^56^. Using HISAT, a fast and sensitive spliced alignment tool based on Bowtie2^57^, the RNA-Seq reads were also mapped to mouse mm9 genome with the default parameters. Mapped reads from biological replicates for ChIP-Seq and RNA-Seq were merged together to facilitate subsequent transcript assembly and quantification.

### *De novo* transcriptome assembly

In this study, for each cell type or developmental stage of tissues, the mapped reads from HISAT were assembled by StringTie which is a highly efficient assembler of RNA-Seq alignments to identify potential transcripts^58^. The assembled transcript files (GTF format) were merged together to form a candidate transcriptome assembly for the novel lincRNA detection^59^.

### The lincRNA detection pipeline

To identify the novel and reliable lincRNAs from mouse ESCs, an optimized analysis pipeline was developed with highly stringent criteria as the following steps: (1) the merged non-redundant transcriptome set was compared with the Ensembl mouse genome annotation, and remained the unannotated transcripts. (2) Size selection. The transcripts with the length ≥200 nt were retained. And for the single exon transcripts whose length more than 200 nt, we only selected the transcripts overlapped with the CAGE peaks within the 1,000 bp of the TSS upstream region. (3) ORF filter. We only kept the transcripts with the ORF length <300 nt^22^. (4) Expression abundance threshold. For the unannotated transcripts passing the length criteria, the transcripts with the FPKM < 1 were removed. (5) Coding potential filter. The coding probability of each remained transcripts was calculated by CPAT^36^, and the transcripts with the score ≤0.44 were retained as the putative lincRNAs from mESCs.

### Training set for TSS proximal region prediction model

We quantify the expression of the known lincRNAs from GENCDOE, and the lincRNAs with the FPKM ≥0.5 were retained. The TSS proximal regions (defined as −1 kb to +0.5 kb with respect to the TSS) were used as the positive training set for model. The 1,500 bp unannotated regions with respect to the sites ±10 kb relative to the lincRNA positive TSSs were used as the negative training set.

### Over-represented motif identification

606 JASPAR TF annotations for position weight matrices (PWMs) were downloaded from the MEME Suite^39^,^40^. FIMO was used to scan the sequences for the 606 TF motifs in the TSS proximal regions of lincRNAs and protein coding transcripts, which is a utility provided by MEME Suite. For each TF motif of the scanning region, it was considered to occur in the corresponding region, if the FIMO q value < 0.05. The over-represented TF motifs were identified for lincRNA TSS positive and negative sets, respectively, by two-tailed Fisher’s exact test with the FDR < 0.01.

### k-mer calculation and represented sequence identification

The term k-mer is often defined as the possible substrings of length k in DNA sequence, which is an essential component for sequence analysis. The probability of the combinations of the four bases (A, T, C, and G) was calculated when k was equal to 2, 3 and 4. 336 K-mer features (K = 2, 3 and 4) of the sequences were calculated by R package seqinr. SCAD (smoothly clipped absolute deviation) penalty^41^,^42^, which is a variable selection method via penalized least squares, was performed to obtain the over-represented k-mer features for distinguishing the lincRNA TSS proximal regions from the negative set with the least error rate by R package penalizedSVM.

### Robust model for predicting lincRNA TSS proximal regions

The RBF SVM machine learning classification algorithm was used to predict the lincRNA TSS proximal regions, in which the RBF Kernel could select solutions smoothly. RBF SVM was implemented by R package e1071 with the parameters of gamma = 1 and cost = 2, which were the best values for the model performance according the tune () function in e1071.

### Model evaluation

10-fold cross-validation was performed for evaluating the model performance, and the assessment measures were calculated, including accuracy, error, sensitivity, specificity and precision. And then, the ROC (Receiver Operating characteristic) curve was drawn to illustrate the performance of the model by plotting the true positive rate against the false positive rate at various threshold settings. The PRC (Precision-Recall) curve was also performed to evaluate the model quality by measuring the relationship between recall and precision at various threshold settings. AUC values of ROC and PRC curves were calculated to estimate the model performance.

### GO annotation of putative lincRNAs through guilt-by-association GSEA approach

The putative lincRNAs expressed in mESC (FPKM ≥ 0.5) mouse ESCs were selected as the candidate lincRNAs for GO annotation. For each candidate lincRNA, a list of ranked protein coding genes was constructed based on the correlation of lincRNAs and all the 11,810 protein coding genes whose expression FPKM value ≥ 0.5 in mouse ESC samples. Using the GO associated gene sets which were obtained from Enrichment Map Gene Sets, including three GO categories molecular function, cellular component and biological process, the enrichment score ES(S) was calculated for the ranked protein coding gene list for the corresponding candidate lincRNA. And then, significant GO terms were identified by the weighted Kolmogorov-Smirnov (KS) test^18^,^51^,^52^. Using 1,000 times permutation, PWER P value was calculated for each GO term, and the GO terms with the FWER P value < 0.05 were considered as the associated GO annotations for the corresponding lincRNA.

## Additional Information

**How to cite this article**: Liu, H. *et al*. Computational identification of putative lincRNAs in mouse embryonic stem cell. *Sci. Rep.* **6**, 34892; doi: 10.1038/srep34892 (2016).

## Supplementary Material

Supplementary Data

Supplementary Table S3

Supplementary Table S4

## Figures and Tables

**Table 1:** Performance assessment of predict models for different features.

Feature	Accuracy	Error rate	Sensitivity	Specificity	Precision	AUC
k-mer	0.79	0.21	0.56	0.90	0.75	0.76
histone	0.83	0.17	0.62	0.93	0.83	0.84
CAGE	0.74	0.26	0.24	0.98	0.87	0.78
Motif	0.86	0.14	0.67	0.95	0.88	0.82
CGI + CpG o/e	0.80	0.20	0.47	0.96	0.85	0.75
Repeat element	0.70	0.30	0.40	0.85	0.57	0.63
All	0.89	0.11	0.80	0.92	0.85	0.90
