# Supplementary material for: Computational identification of putative lincRNAs in mouse embryonic stem cell
Source: Sci Rep. 2016 Oct 7;6:34892. doi: 10.1038/srep34892 (PMC5054606; doi:10.1038/srep34892)
Supplement: Supplementary Data [file srep34892-s1.doc]

**Supplementary data for**

**Computational identification of putative lincRNAs in mouse embryonic stem cell**

Hui Liu, Jie Lyu, Hongbo Liu, Yang Gao, Jing Guo, Hongjuan He, Zhengbin Han, Yan Zhang and Qiong Wu

**Supplementary Figures**


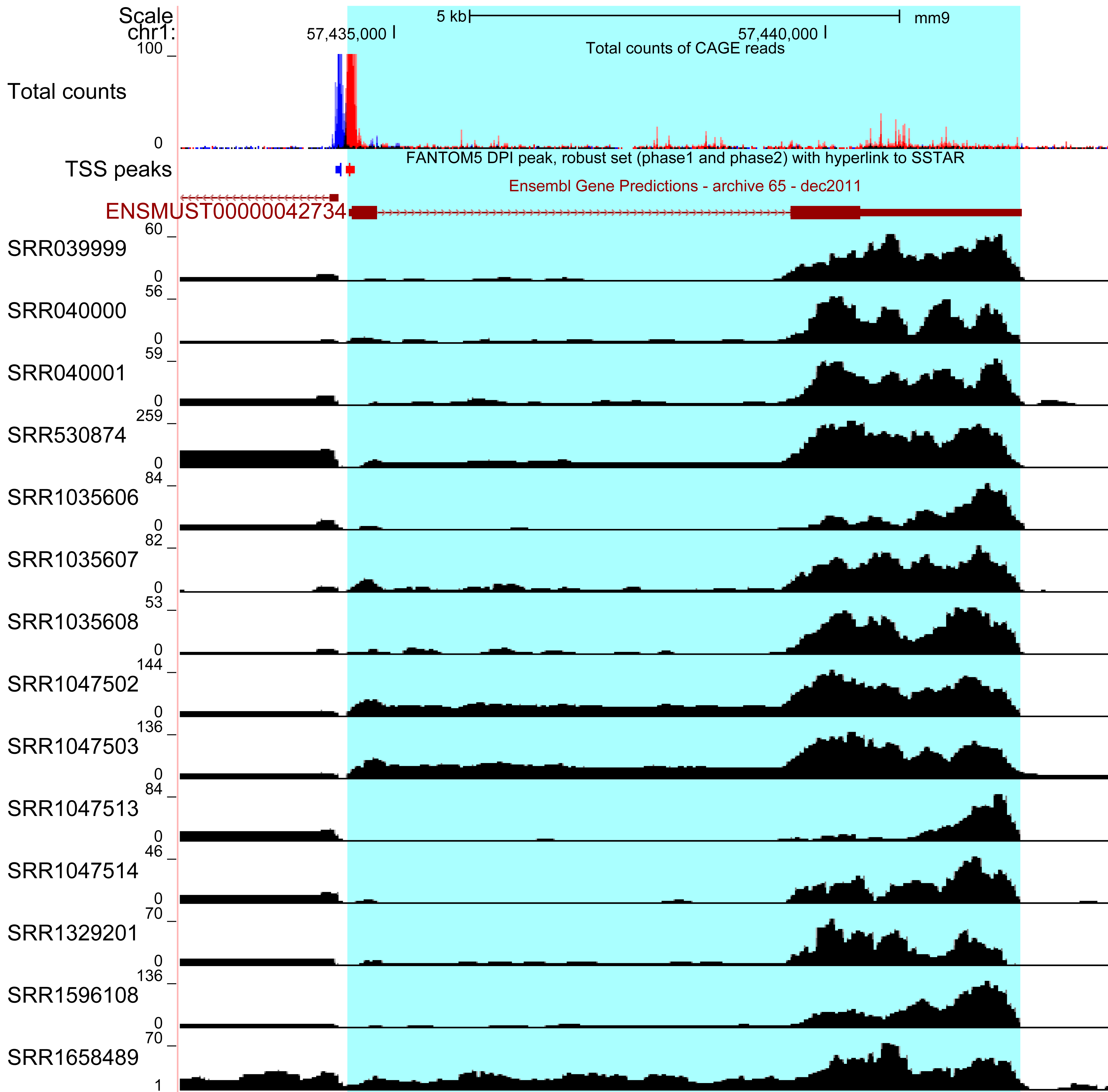


Supplementary Figure S1. An example of a known protein coding transcript ENSMUST00000042734. UCSC Genome Browser tracks showing 14 RNA-Seq reads from mouse ESCs for this transcript. Data for each RNA-Seq sample are shown as a ‘wiggle’ format. Genome coordinates are from the NCBI37/mm9 assembly of the mouse genome.


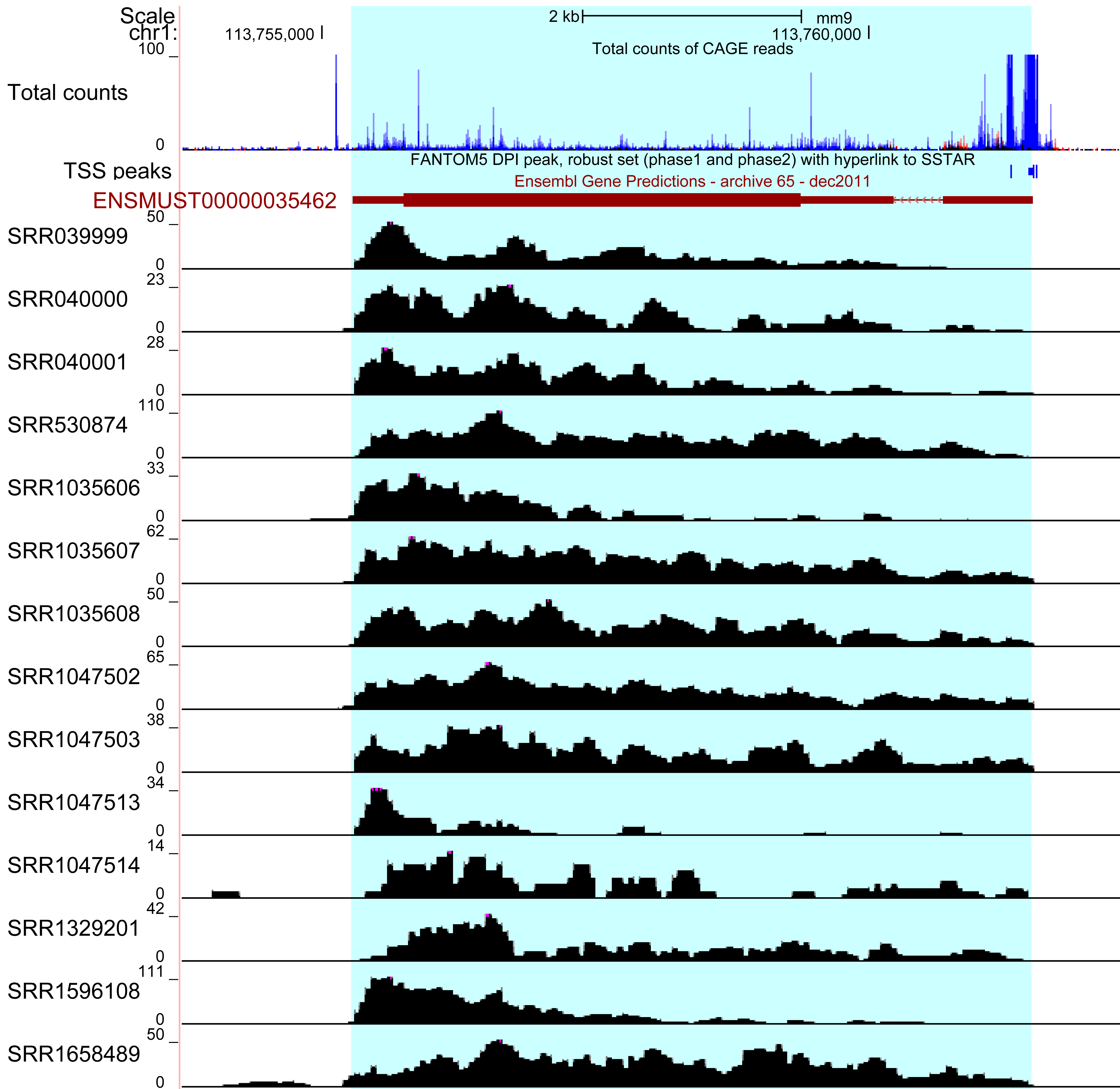


Supplementary Figure S2. An example of a known protein coding transcript ENSMUST00000 035462. UCSC Genome Browser tracks showing 14 RNA-Seq reads from mouse ESCs for this transcript. Data for each RNA-Seq sample are shown as a ‘wiggle’ format. Genome coordinates are from the NCBI37/mm9 assembly of the mouse genome.


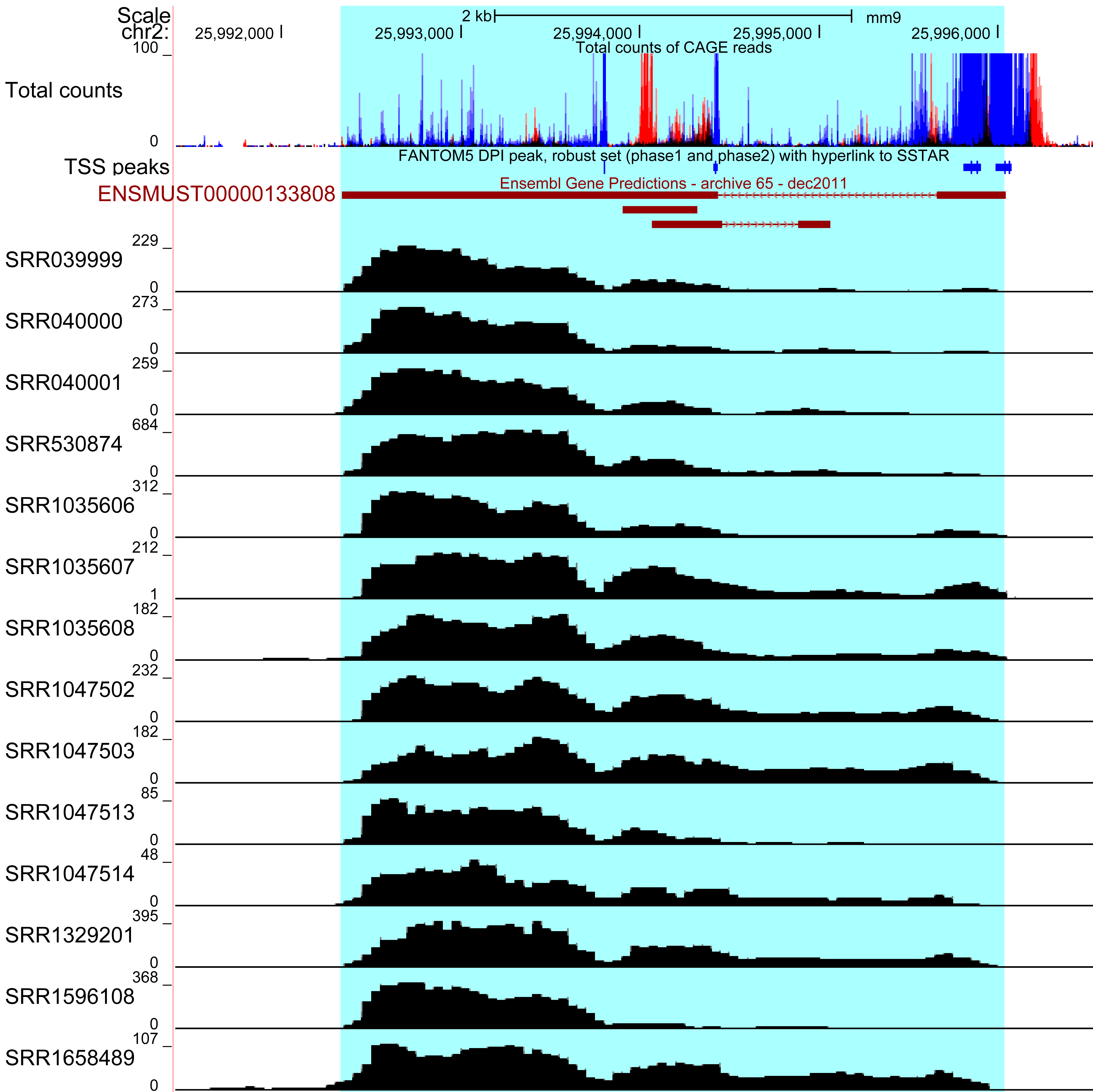


Supplementary Figure S3. An example of a known lincRNA transcript ENSMUST00000133808. UCSC Genome Browser tracks showing 14 RNA-Seq reads from mouse ESCs for this transcript. Data for each RNA-Seq sample are shown as a ‘wiggle’ format. Genome coordinates are from the NCBI37/mm9 assembly of the mouse genome.


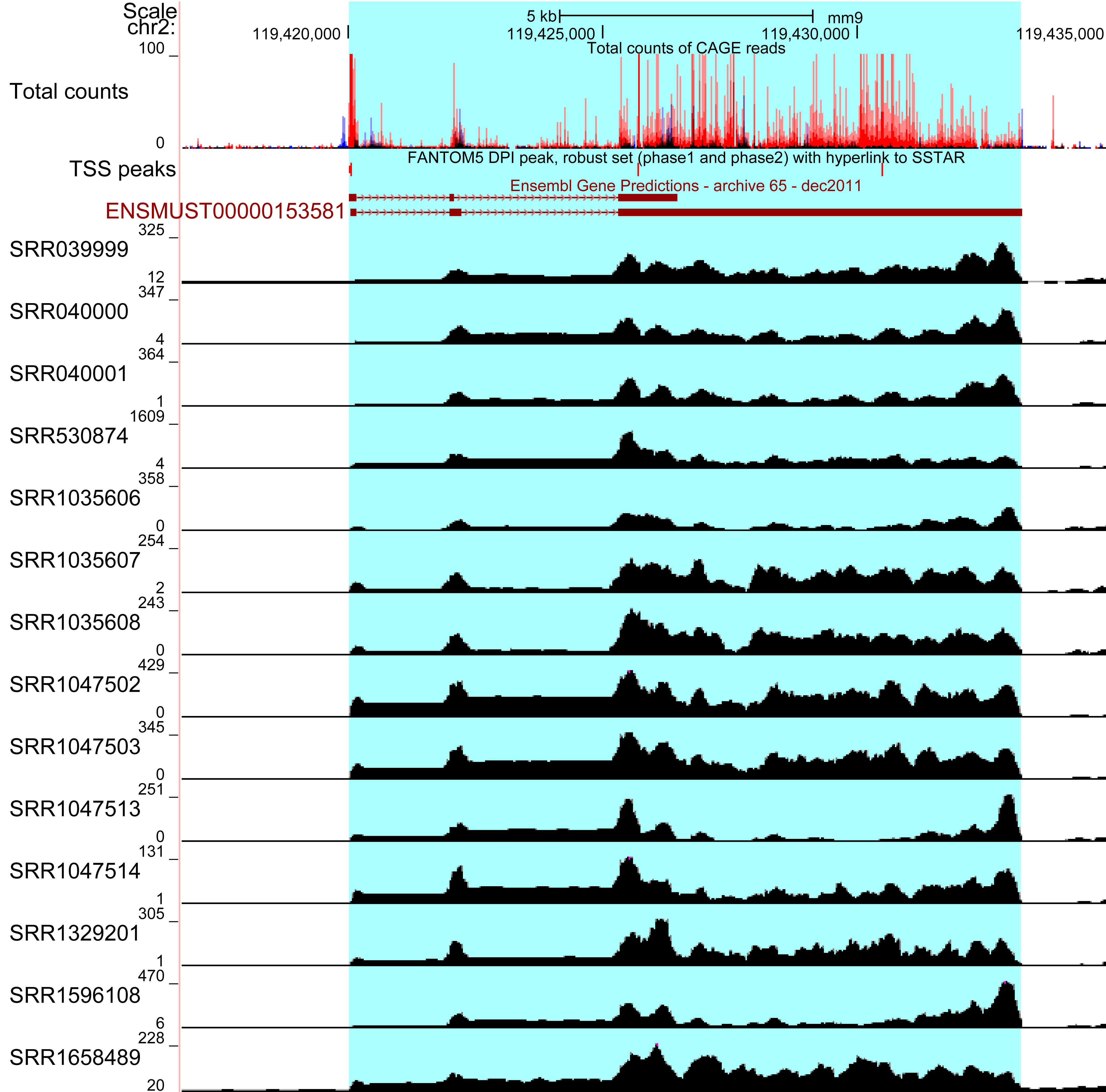


Supplementary Figure S4. An example of a known lincRNA transcript ENSMUST00000 153581. UCSC Genome Browser tracks showing 14 RNA-Seq reads from mouse ESCs for this transcript. Data for each RNA-Seq sample are shown as a ‘wiggle’ format. Genome coordinates are from the NCBI37/mm9 assembly of the mouse genome.


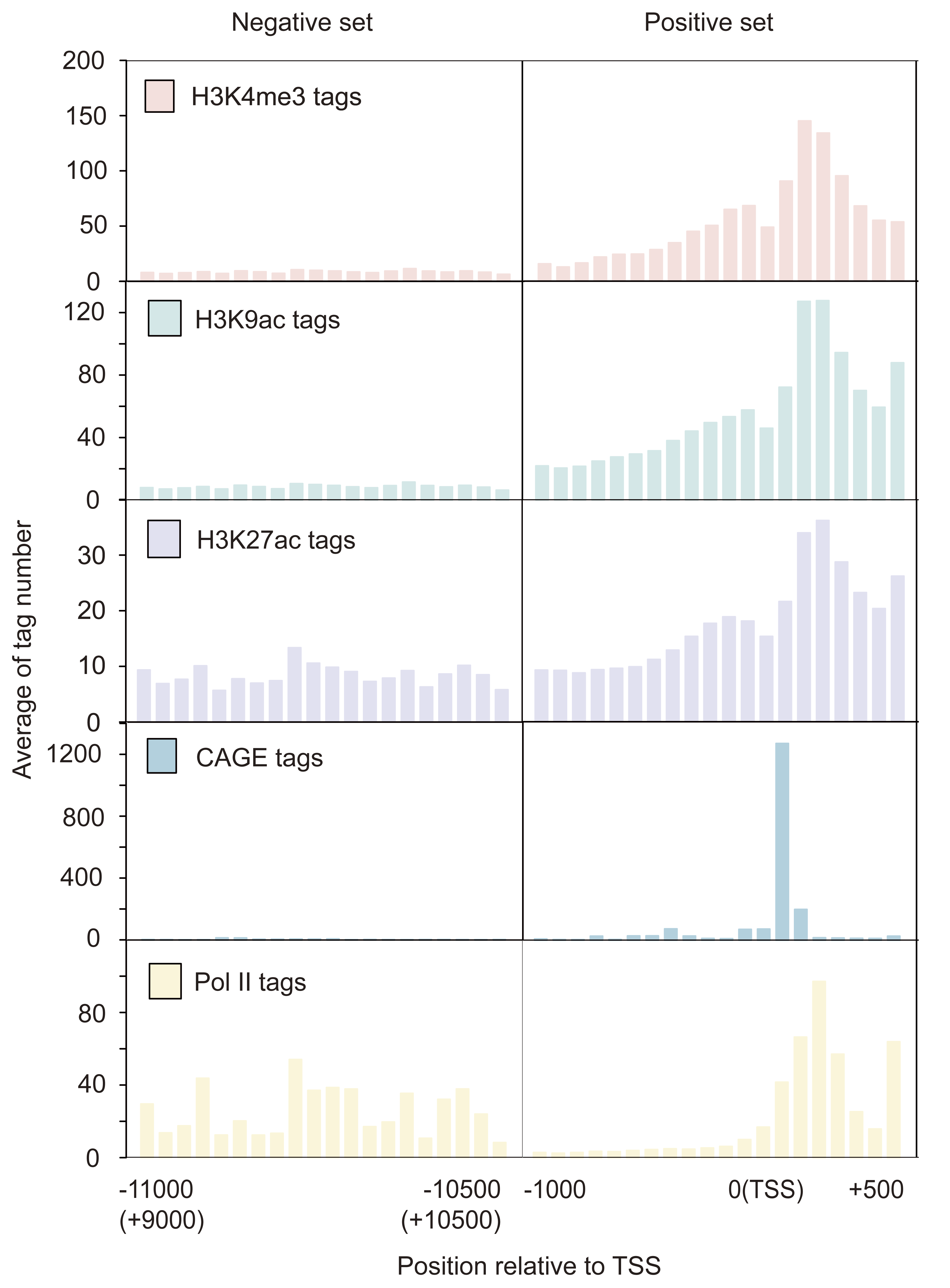


Supplementary Figure S5. The comparison of the chromatin modifications of positive and negative sets. The average tag count distributions of H3k4me3, H3k9ac, H3k27ac, CAGE and Pol II modifications are shown for positive and negative sets.


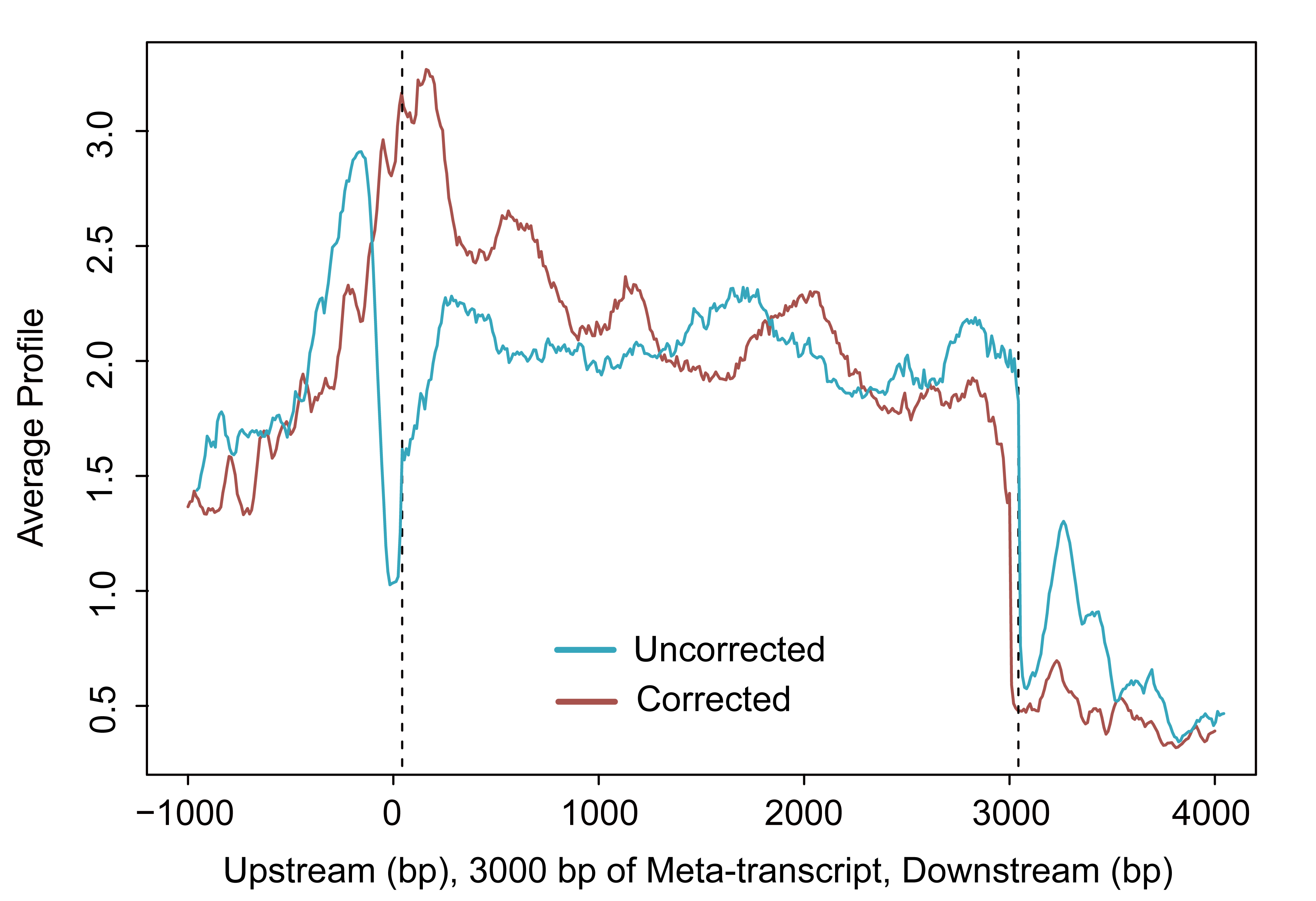


Supplementary Figure S6. Average profile of CAGE signals for 1293 putative lincRNA transcript bodies before and after correction of 5' ends. The size of all the transcript bodies are normalized the same length 3000 bp for comparison. Red line corresponds to lincRNAs before correction, and blue line corresponds to lincNRAs after correction.


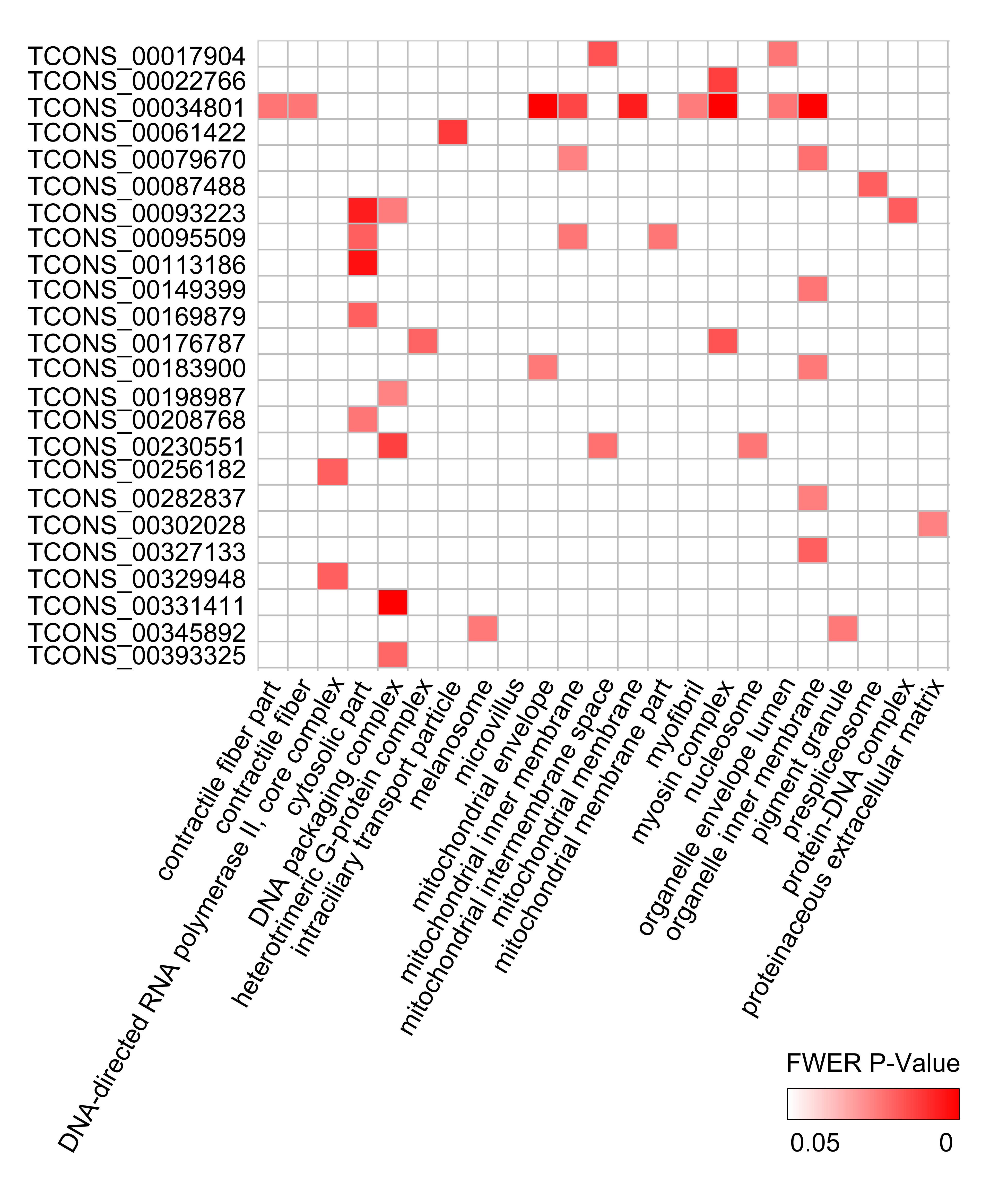


Supplementary Figure S7. The GO cellular components of the putative lincRNAs annotated through guilt-by association method.


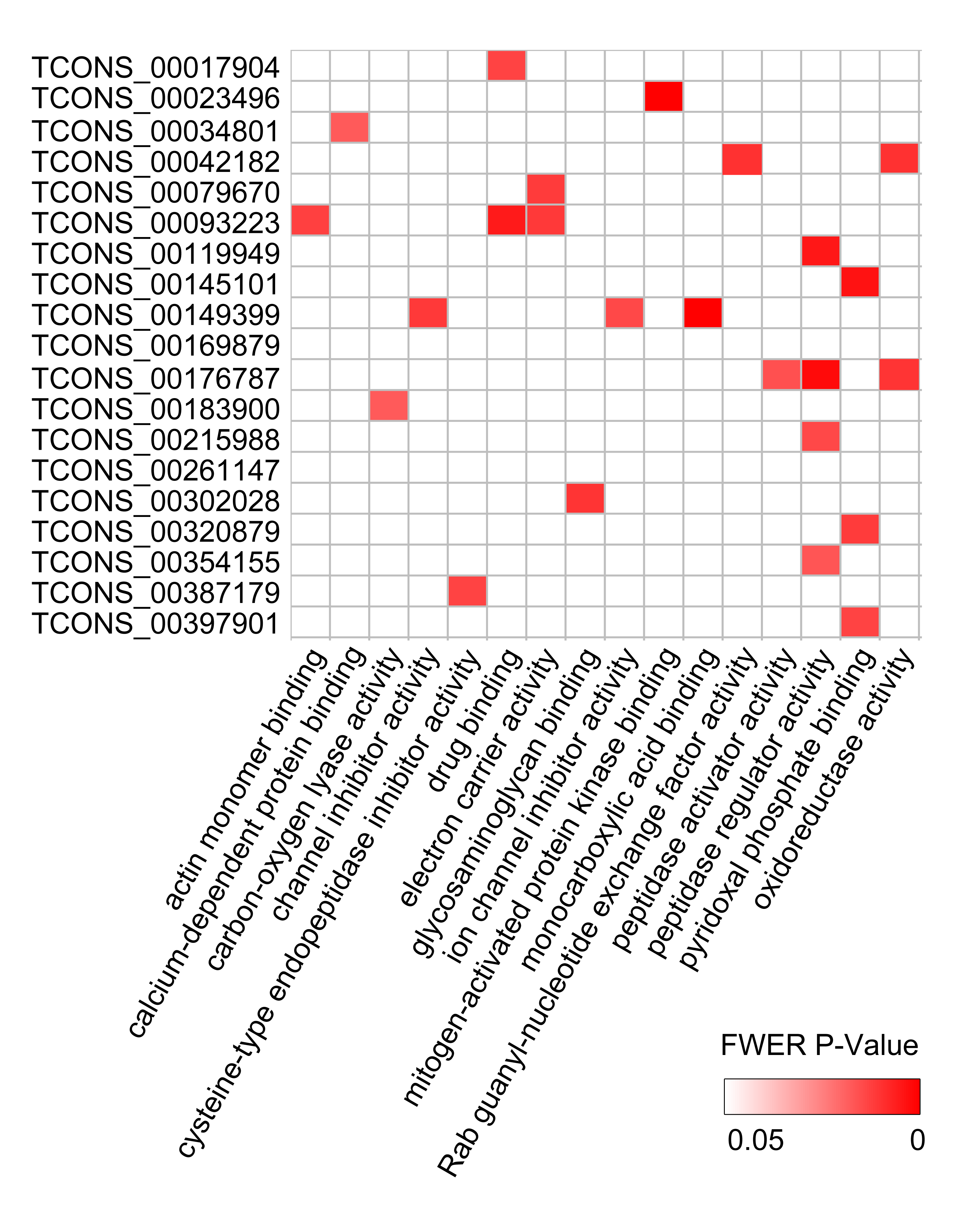


Supplementary Figure S8. The GO molecular functions of the putative lincRNAs annotated through guilt-by association method.

**Supplementary Tables**

Supplementary Table S1. RNA-Seq data used in this study.

| SRA ID | tissue or cell type | RNA | GEO ID | layout | read length | development stage | PubMed ID | reference |
| --- | --- | --- | --- | --- | --- | --- | --- | --- |
| SRR039999 | mESCs | polyA+ RNA | GSE20851 | PAIRED | 76 | E0 | 20436462 | [^60^](#_ENREF_1) |
| SRR040000 | mESCs | polyA+ RNA | GSE20851 | PAIRED | 76 | E0 | 20436462 | [^60^](#_ENREF_1) |
| SRR040001 | mESCs | polyA+ RNA | GSE20851 | PAIRED | 76 | E0 | 20436462 | [^60^](#_ENREF_1) |
| SRR530874 | mESCs | total RNA | GSE39513 | PAIRED | 100 | E0 | 23101626 | [^61^](#_ENREF_2) |
| SRR1035606 | mESCs | total RNA | GSE55698 | PAIRED | 51 | E0 | 24856970 | [^62^](#_ENREF_3) |
| SRR1035607 | mESCs | total RNA | GSE55698 | PAIRED | 51 | E0 | 24856970 | [^62^](#_ENREF_3) |
| SRR1035608 | mESCs | total RNA | GSE55698 | PAIRED | 51 | E0 | 24856970 | [^62^](#_ENREF_3) |
| SRR1047502 | mESCs | total RNA | GSE53212 | PAIRED | 101 | E0 | 24527385 | [^63^](#_ENREF_4) |
| SRR1047503 | mESCs | total RNA | GSE53212 | PAIRED | 101 | E0 | 24527385 | [^63^](#_ENREF_4) |
| SRR1047513 | mESCs | total RNA | GSE53212 | PAIRED | 100 | E0 | 24527385 | [^63^](#_ENREF_4) |
| SRR1047514 | mESCs | total RNA | GSE53212 | PAIRED | 100 | E0 | 24527385 | [^63^](#_ENREF_4) |
| SRR1329201 | mESCs | polyA+ RNA | GSE58206 | PAIRED | 101 | E0 | 25857206 | [^64^](#_ENREF_5) |
| SRR1596108 | mESCs | polyA+ RNA | GSE61997 | PAIRED | 101 | E0 | 25569111 | [^65^](#_ENREF_6) |
| SRR1658489 | mESCs | total RNA | GSE63523 | PAIRED | 100 | E0 | 25607992 | [^66^](#_ENREF_7) |
| SRR689238 | 2-cell blastomere | polyA+ RNA | GSE44183 | PAIRED | 90 | E0.5 | 23892778 | [^67^](#_ENREF_8) |
| SRR689239 | 2-cell blastomere | polyA+ RNA | GSE44183 | PAIRED | 90 | E0.5 | 23892778 | [^67^](#_ENREF_8) |
| SRR689240 | 2-cell blastomere | polyA+ RNA | GSE44183 | PAIRED | 90 | E0.5 | 23892778 | [^67^](#_ENREF_8) |
| SRR689241 | 4-cell blastomere | polyA+ RNA | GSE44183 | PAIRED | 90 | E1.5 | 23892778 | [^67^](#_ENREF_8) |
| SRR689242 | 4-cell blastomere | polyA+ RNA | GSE44183 | PAIRED | 90 | E1.5 | 23892778 | [^67^](#_ENREF_8) |
| SRR689243 | 4-cell blastomere | polyA+ RNA | GSE44183 | PAIRED | 90 | E1.5 | 23892778 | [^67^](#_ENREF_8) |
| SRR689244 | 8-cell blastomere | polyA+ RNA | GSE44183 | PAIRED | 90 | E2.5 | 23892778 | [^67^](#_ENREF_8) |
| SRR689245 | 8-cell blastomere | polyA+ RNA | GSE44183 | PAIRED | 90 | E2.5 | 23892778 | [^67^](#_ENREF_8) |
| SRR689246 | 8-cell blastomere | polyA+ RNA | GSE44183 | PAIRED | 90 | E2.5 | 23892778 | [^67^](#_ENREF_8) |
| SRR689247 | morula | polyA+ RNA | GSE44183 | PAIRED | 90 | E3.0 | 23892778 | [^67^](#_ENREF_8) |
| SRR689248 | morula | polyA+ RNA | GSE44183 | PAIRED | 90 | E3.0 | 23892778 | [^67^](#_ENREF_8) |
| SRR689249 | morula | polyA+ RNA | GSE44183 | PAIRED | 90 | E3.0 | 23892778 | [^67^](#_ENREF_8) |
| SRR1652561 | whole embryos | total RNA | GSE60334 | PAIRED | 76 | E8.5 | 26576615 | [^68^](#_ENREF_9) |
| SRR1652562 | whole embryos | total RNA | GSE60334 | PAIRED | 76 | E8.5 | 26576615 | [^68^](#_ENREF_9) |
| SRR1652563 | whole embryos | total RNA | GSE60334 | PAIRED | 76 | E8.5 | 26576615 | [^68^](#_ENREF_9) |
| SRR851934 | whole embryos | total RNA | GSE47033 | PAIRED | 74 | E10.5 | 24709821 | [^69^](#_ENREF_10) |
| SRR851935 | whole embryos | total RNA | GSE47033 | PAIRED | 74 | E10.5 | 24709821 | [^69^](#_ENREF_10) |
| SRR948344 | Embryonic Telencephalon | total RNA | GSE49581 | PAIRED | 101 | E13.5 | 24381249 | [^70^](#_ENREF_11) |
| SRR948345 | Embryonic Telencephalon | total RNA | GSE49581 | PAIRED | 101 | E13.5 | 24381249 | [^70^](#_ENREF_11) |
| SRR948346 | Embryonic Telencephalon | total RNA | GSE49581 | PAIRED | 101 | E13.5 | 24381249 | [^70^](#_ENREF_11) |
| SRR948347 | Embryonic Telencephalon | total RNA | GSE49581 | PAIRED | 101 | E13.5 | 24381249 | [^70^](#_ENREF_11) |
| SRR948348 | Embryonic Telencephalon | total RNA | GSE49581 | PAIRED | 100 | E15.5 | 24381249 | [^70^](#_ENREF_11) |
| SRR948349 | Embryonic Telencephalon | total RNA | GSE49581 | PAIRED | 100 | E15.5 | 24381249 | [^70^](#_ENREF_11) |
| SRR948350 | Embryonic Telencephalon | total RNA | GSE49581 | PAIRED | 100 | E15.5 | 24381249 | [^70^](#_ENREF_11) |
| SRR948351 | Embryonic Telencephalon | total RNA | GSE49581 | PAIRED | 100 | E15.5 | 24381249 | [^70^](#_ENREF_11) |
| SRR948352 | Embryonic Telencephalon | total RNA | GSE49581 | PAIRED | 100 | E15.5 | 24381249 | [^70^](#_ENREF_11) |
| SRR948353 | Whole Brain | total RNA | GSE49581 | PAIRED | 101 | P7 | 24381249 | [^70^](#_ENREF_11) |
| SRR948354 | Whole Brain | total RNA | GSE49581 | PAIRED | 101 | P7 | 24381249 | [^70^](#_ENREF_11) |
| SRR948355 | Whole Brain | total RNA | GSE49581 | PAIRED | 101 | P7 | 24381249 | [^70^](#_ENREF_11) |
| SRR948356 | Whole Brain | total RNA | GSE49581 | PAIRED | 101 | P7 | 24381249 | [^70^](#_ENREF_11) |
| SRR948336 | Embryonic Brain | total RNA | GSE49581 | PAIRED | 101 | E18.5 | 24381249 | [^70^](#_ENREF_11) |
| SRR948337 | Embryonic Brain | total RNA | GSE49581 | PAIRED | 101 | E18.5 | 24381249 | [^70^](#_ENREF_11) |
| SRR948338 | Embryonic Brain | total RNA | GSE49581 | PAIRED | 101 | E18.5 | 24381249 | [^70^](#_ENREF_11) |
| SRR948339 | Embryonic Brain | total RNA | GSE49581 | PAIRED | 101 | E18.5 | 24381249 | [^70^](#_ENREF_11) |
| SRR948340 | Embryonic Lung | total RNA | GSE49581 | PAIRED | 101 | E14.5 | 24381249 | [^70^](#_ENREF_11) |
| SRR948341 | Embryonic Lung | total RNA | GSE49581 | PAIRED | 101 | E14.5 | 24381249 | [^70^](#_ENREF_11) |
| SRR948342 | Embryonic Lung | total RNA | GSE49581 | PAIRED | 101 | E14.5 | 24381249 | [^70^](#_ENREF_11) |
| SRR948343 | Embryonic Lung | total RNA | GSE49581 | PAIRED | 101 | E14.5 | 24381249 | [^70^](#_ENREF_11) |
| SRR496249 | mESCs | polyA+ RNA | GSE36026 | SINGLE | 30 | E0 | 25409824 | [^71^](#_ENREF_12) |

Supplementary Table S2. ChIP-Seq data used in this study.

| SRA ID | tissue or cell type | antibody | GEO ID | layout | read length | development stage | PubMed ID | reference |
| --- | --- | --- | --- | --- | --- | --- | --- | --- |
| SRR317222 | mESCs | H3k4me3 | GSE49847 | SINGLE | 36 | E0 | 25409824 | [^71^](#_ENREF_12) |
| SRR317223 | mESCs | H3k4me3 | GSE49847 | SINGLE | 36 | E0 | 25409824 | [^71^](#_ENREF_12) |
| SRR566895 | mESCs | H3k9ac | GSE49847 | SINGLE | 50 | E0 | 25409824 | [^71^](#_ENREF_12) |
| SRR566896 | mESCs | H3k9ac | GSE49847 | SINGLE | 50 | E0 | 25409824 | [^71^](#_ENREF_12) |
| SRR566839 | mESCs | H3k27ac | GSE49847 | SINGLE | 36 | E0 | 25409824 | [^71^](#_ENREF_12) |
| SRR566840 | mESCs | H3k27ac | GSE49847 | SINGLE | 36 | E0 | 25409824 | [^71^](#_ENREF_12) |
| SRR489721 | mESCs | PolII | GSE49847 | SINGLE | 36 | E0 | 25409824 | [^71^](#_ENREF_12) |
| SRR489722 | mESCs | PolII | GSE49847 | SINGLE | 36 | E0 | 25409824 | [^71^](#_ENREF_12) |
| SRR006789 | mESCs | H3k4me3 | GSE12241 | SINGLE | 27 | E0 | 17603471;19946270 |  |
| SRR006828 | mESCs | H3k4me3 | GSE12241 | SINGLE | 27 | E0 | 17603471;19946270 |  |
| SRR006829 | mESCs | H3k4me3 | GSE12241 | SINGLE | 27 | E0 | 17603471;19946270 |  |
| SRR006830 | mESCs | H3k4me3 | GSE12241 | SINGLE | 27 | E0 | 17603471;19946270 |  |
| SRR006831 | mESCs | H3k4me3 | GSE12241 | SINGLE | 36 | E0 | 17603471;19946270 |  |
| SRR006832 | mESCs | H3k4me3 | GSE12241 | SINGLE | 36 | E0 | 17603471;19946270 |  |
| SRR572714 | mESCs | H3K27ac | GSE40951 | SINGLE | 48 | E0 | 23085715;24812327 |  |
| SRR572715 | mESCs | H3K9ac | GSE40951 | SINGLE | 48 | E0 | 23085715;24812327 |  |

Supplementary Table S5. GO BP annotations of putative lincRNAs by guilt-by-association method.

| probe.ID | GO.name | GO.ID | pos.or.neg | NES | FWER p-val |
| --- | --- | --- | --- | --- | --- |
| TCONS_00393325 | PROTEIN PEPTIDYL-PROLYL ISOMERIZATION | GO:0000413 | neg | -2.2345042 | 0.008 |
| TCONS_00281234 | HYDROGEN TRANSPORT | GO:0006818 | pos | 2.1320314 | 0.033 |
| TCONS_00281234 | PROTON TRANSPORT | GO:0015992 | pos | 2.1320314 | 0.033 |
| TCONS_00087488 | TRANSCRIPTION FROM RNA POLYMERASE III PROMOTER | GO:0006383 | pos | 2.2170007 | 0.012 |
| TCONS_00183900 | PEPTIDE METABOLIC PROCESS | GO:0006518 | pos | 2.2539954 | 0.027 |
| TCONS_00183900 | GLYCOSYL COMPOUND BIOSYNTHETIC PROCESS | GO:1901659 | pos | 2.245757 | 0.03 |
| TCONS_00183900 | CELLULAR AMIDE METABOLIC PROCESS | GO:0043603 | pos | 2.2440693 | 0.03 |
| TCONS_00183900 | RIBONUCLEOSIDE BIOSYNTHETIC PROCESS | GO:0042455 | pos | 2.2410069 | 0.033 |
| TCONS_00183900 | PURINE RIBONUCLEOTIDE METABOLIC PROCESS | GO:0009150 | pos | 2.2344716 | 0.036 |
| TCONS_00183900 | NUCLEOSIDE BIOSYNTHETIC PROCESS | GO:0009163 | pos | 2.228153 | 0.039 |
| TCONS_00183900 | RIBOSE PHOSPHATE METABOLIC PROCESS | GO:0019693 | pos | 2.2204554 | 0.047 |
| TCONS_00183900 | RIBONUCLEOTIDE METABOLIC PROCESS | GO:0009259 | pos | 2.2197547 | 0.047 |
| TCONS_00023496 | NEGATIVE REGULATION OF CELL CYCLE G2/M PHASE TRANSITION | GO:1902750 | pos | 2.2203703 | 0.002 |
| TCONS_00023496 | NEGATIVE REGULATION OF G2/M TRANSITION OF MITOTIC CELL CYCLE | GO:0010972 | pos | 2.1577852 | 0.026 |
| TCONS_00230551 | CHROMATIN ASSEMBLY | GO:0031497 | pos | 2.3849459 | 0.047 |
| TCONS_00230551 | CHROMATIN ASSEMBLY OR DISASSEMBLY | GO:0006333 | pos | 2.376652 | 0.047 |
| TCONS_00230551 | NUCLEOSOME ASSEMBLY | GO:0006334 | pos | 2.362986 | 0.047 |
| TCONS_00230551 | NUCLEOSOME ORGANIZATION | GO:0034728 | pos | 2.3122878 | 0.047 |
| TCONS_00230551 | PROTEIN HETEROTETRAMERIZATION | GO:0051290 | pos | 2.2903683 | 0.048 |
| TCONS_00230551 | NEGATIVE REGULATION OF STRIATED MUSCLE CELL APOPTOTIC PROCESS | GO:0010664 | neg | -2.1337383 | 0.034 |
| TCONS_00302028 | TRANSCRIPTION FROM RNA POLYMERASE III PROMOTER | GO:0006383 | pos | 2.138822 | 0.024 |
| TCONS_00339729 | PROTEIN PEPTIDYL-PROLYL ISOMERIZATION | GO:0000413 | neg | -2.3188953 | 0 |
| TCONS_00093223 | CHROMATIN ASSEMBLY OR DISASSEMBLY | GO:0006333 | pos | 2.4868724 | 0 |
| TCONS_00093223 | CHROMATIN ASSEMBLY | GO:0031497 | pos | 2.4848561 | 0 |
| TCONS_00093223 | NUCLEOSOME ORGANIZATION | GO:0034728 | pos | 2.4309907 | 0 |
| TCONS_00093223 | NUCLEOSOME ASSEMBLY | GO:0006334 | pos | 2.4273295 | 0 |
| TCONS_00093223 | DNA PACKAGING | GO:0006323 | pos | 2.388023 | 0.001 |
| TCONS_00093223 | PROTEIN-DNA COMPLEX ASSEMBLY | GO:0065004 | pos | 2.3822458 | 0.001 |
| TCONS_00093223 | PROTEIN-DNA COMPLEX SUBUNIT ORGANIZATION | GO:0071824 | pos | 2.327437 | 0.003 |
| TCONS_00093223 | NEGATIVE REGULATION OF STRIATED MUSCLE CELL APOPTOTIC PROCESS | GO:0010664 | neg | -2.4501872 | 0 |
| TCONS_00093223 | SYNAPSIS | GO:0007129 | neg | -2.161744 | 0.018 |
| TCONS_00198987 | PROTEIN PEPTIDYL-PROLYL ISOMERIZATION | GO:0000413 | neg | -2.1456172 | 0.014 |
| TCONS_00126514 | PROTEIN PEPTIDYL-PROLYL ISOMERIZATION | GO:0000413 | neg | -2.1210442 | 0.037 |
| TCONS_00081732 | PEPTIDYL-LYSINE TRIMETHYLATION | GO:0018023 | neg | -2.194318 | 0.031 |
| TCONS_00393321 | PROTEIN PEPTIDYL-PROLYL ISOMERIZATION | GO:0000413 | neg | -2.1539068 | 0.027 |
| TCONS_00343141 | PROTEIN PEPTIDYL-PROLYL ISOMERIZATION | GO:0000413 | neg | -2.243879 | 0.002 |
| TCONS_00034801 | CELLULAR AMIDE METABOLIC PROCESS | GO:0043603 | pos | 2.295032 | 0.015 |
| TCONS_00034801 | RIBOSE PHOSPHATE METABOLIC PROCESS | GO:0019693 | pos | 2.2883997 | 0.018 |
| TCONS_00034801 | MITOCHONDRIAL TRANSPORT | GO:0006839 | pos | 2.284874 | 0.019 |
| TCONS_00034801 | GLYCOSYL COMPOUND METABOLIC PROCESS | GO:1901657 | pos | 2.2804594 | 0.021 |
| TCONS_00034801 | PURINE RIBONUCLEOTIDE METABOLIC PROCESS | GO:0009150 | pos | 2.2732983 | 0.022 |
| TCONS_00034801 | PURINE NUCLEOSIDE METABOLIC PROCESS | GO:0042278 | pos | 2.271679 | 0.022 |
| TCONS_00034801 | RIBONUCLEOTIDE METABOLIC PROCESS | GO:0009259 | pos | 2.2658947 | 0.024 |
| TCONS_00034801 | PURINE NUCLEOTIDE METABOLIC PROCESS | GO:0006163 | pos | 2.264957 | 0.024 |
| TCONS_00034801 | RIBONUCLEOSIDE METABOLIC PROCESS | GO:0009119 | pos | 2.2648883 | 0.024 |
| TCONS_00034801 | PEPTIDE METABOLIC PROCESS | GO:0006518 | pos | 2.2610645 | 0.026 |
| TCONS_00034801 | ISOPRENOID METABOLIC PROCESS | GO:0006720 | pos | 2.2580245 | 0.026 |
| TCONS_00034801 | NUCLEOSIDE METABOLIC PROCESS | GO:0009116 | pos | 2.2477052 | 0.03 |
| TCONS_00034801 | PURINE RIBONUCLEOSIDE METABOLIC PROCESS | GO:0046128 | pos | 2.2376368 | 0.034 |
| TCONS_00034801 | PURINE NUCLEOSIDE MONOPHOSPHATE METABOLIC PROCESS | GO:0009126 | pos | 2.2203882 | 0.044 |
| TCONS_00034801 | PURINE RIBONUCLEOSIDE MONOPHOSPHATE METABOLIC PROCESS | GO:0009167 | pos | 2.2203882 | 0.044 |
| TCONS_00034801 | PURINE-CONTAINING COMPOUND METABOLIC PROCESS | GO:0072521 | pos | 2.2049453 | 0.049 |
| TCONS_00034801 | AMIDE BIOSYNTHETIC PROCESS | GO:0043604 | pos | 2.2046351 | 0.049 |
| TCONS_00192902 | PROTEIN PEPTIDYL-PROLYL ISOMERIZATION | GO:0000413 | neg | -2.235367 | 0.004 |
| TCONS_00025501 | DNA METHYLATION | GO:0006306 | neg | -2.1906095 | 0.026 |
| TCONS_00025501 | DNA ALKYLATION | GO:0006305 | neg | -2.1906095 | 0.026 |
| TCONS_00025501 | DNA MODIFICATION | GO:0006304 | neg | -2.1486359 | 0.046 |
| TCONS_00142666 | PROTEIN PEPTIDYL-PROLYL ISOMERIZATION | GO:0000413 | neg | -2.1062722 | 0.03 |

Supplementary Table S6. GO CC annotations of putative lincRNAs by guilt-by-association method.

| probe.ID | GO.name | GO.ID | pos.or.neg | NES | FWER p-val |
| --- | --- | --- | --- | --- | --- |
| TCONS_00017904 | MITOCHONDRIAL INTERMEMBRANE SPACE | GO:0005758 | neg | -2.11115 | 0.015 |
| TCONS_00017904 | ORGANELLE ENVELOPE LUMEN | GO:0031970 | neg | -2.04604 | 0.035 |
| TCONS_00022766 | MYOSIN COMPLEX | GO:0016459 | pos | 2.138526 | 0.009 |
| TCONS_00093223 | PROTEIN-DNA COMPLEX | GO:0032993 | pos | 2.264766 | 0.019 |
| TCONS_00093223 | DNA PACKAGING COMPLEX | GO:0044815 | pos | 2.180899 | 0.041 |
| TCONS_00093223 | CYTOSOLIC PART | GO:0044445 | neg | -2.2297 | 0.004 |
| TCONS_00034801 | MYOSIN COMPLEX | GO:0016459 | pos | 2.356777 | 0.002 |
| TCONS_00034801 | MITOCHONDRIAL ENVELOPE | GO:0005740 | pos | 2.344277 | 0.002 |
| TCONS_00034801 | ORGANELLE INNER MEMBRANE | GO:0019866 | pos | 2.343542 | 0.002 |
| TCONS_00034801 | MITOCHONDRIAL MEMBRANE | GO:0031966 | pos | 2.320561 | 0.004 |
| TCONS_00034801 | MITOCHONDRIAL INNER MEMBRANE | GO:0005743 | pos | 2.269824 | 0.011 |
| TCONS_00034801 | SARCOMERE | GO:0030017 | pos | 2.244075 | 0.018 |
| TCONS_00034801 | CONTRACTILE FIBER PART | GO:0044449 | pos | 2.17099 | 0.033 |
| TCONS_00034801 | ORGANELLE ENVELOPE LUMEN | GO:0031970 | pos | 2.162555 | 0.035 |
| TCONS_00034801 | CONTRACTILE FIBER | GO:0043292 | pos | 2.161857 | 0.036 |
| TCONS_00034801 | MYOFIBRIL | GO:0030016 | pos | 2.146007 | 0.04 |
| TCONS_00061422 | INTRACILIARY TRANSPORT PARTICLE | GO:0030990 | neg | -2.04804 | 0.008 |
| TCONS_00079670 | ORGANELLE INNER MEMBRANE | GO:0019866 | pos | 2.096085 | 0.029 |
| TCONS_00079670 | MITOCHONDRIAL INNER MEMBRANE | GO:0005743 | pos | 2.057482 | 0.045 |
| TCONS_00087488 | PRESPLICEOSOME | GO:0071010 | pos | 2.162897 | 0.021 |
| TCONS_00095509 | CYTOSOLIC PART | GO:0044445 | pos | 2.122798 | 0.02 |
| TCONS_00095509 | MITOCHONDRIAL INNER MEMBRANE | GO:0005743 | pos | 2.083156 | 0.036 |
| TCONS_00095509 | MITOCHONDRIAL MEMBRANE PART | GO:0044455 | pos | 2.080776 | 0.036 |
| TCONS_00113186 | CYTOSOLIC PART | GO:0044445 | neg | -2.23999 | 0.003 |
| TCONS_00149399 | ORGANELLE INNER MEMBRANE | GO:0019866 | pos | 2.045578 | 0.034 |
| TCONS_00176787 | MYOSIN COMPLEX | GO:0016459 | pos | 2.184373 | 0.015 |
| TCONS_00176787 | HETEROTRIMERIC G-PROTEIN COMPLEX | GO:0005834 | pos | 2.158593 | 0.023 |
| TCONS_00183900 | ORGANELLE INNER MEMBRANE | GO:0019866 | pos | 2.159782 | 0.038 |
| TCONS_00183900 | MITOCHONDRIAL ENVELOPE | GO:0005740 | pos | 2.151431 | 0.039 |
| TCONS_00198987 | DNA PACKAGING COMPLEX | GO:0044815 | pos | 2.375917 | 0.048 |
| TCONS_00208768 | CYTOSOLIC PART | GO:0044445 | pos | 2.059196 | 0.037 |
| TCONS_00230551 | DNA PACKAGING COMPLEX | GO:0044815 | pos | 2.318613 | 0.01 |
| TCONS_00230551 | NUCLEOSOME | GO:0000786 | pos | 2.246847 | 0.035 |
| TCONS_00230551 | MITOCHONDRIAL INTERMEMBRANE SPACE | GO:0005758 | neg | -2.0161 | 0.031 |
| TCONS_00256182 | DNA-DIRECTED RNA POLYMERASE II, CORE COMPLEX | GO:0005665 | pos | 2.151569 | 0.02 |
| TCONS_00282837 | ORGANELLE INNER MEMBRANE | GO:0019866 | pos | 2.055077 | 0.043 |
| TCONS_00302028 | PROTEINACEOUS EXTRACELLULAR MATRIX | GO:0005578 | neg | -2.04252 | 0.046 |
| TCONS_00327133 | ORGANELLE INNER MEMBRANE | GO:0019866 | pos | 2.109561 | 0.02 |
| TCONS_00329948 | DNA-DIRECTED RNA POLYMERASE II, CORE COMPLEX | GO:0005665 | pos | 2.131175 | 0.026 |
| TCONS_00331411 | DNA PACKAGING COMPLEX | GO:0044815 | pos | 2.561342 | 0.002 |
| TCONS_00169879 | CYTOSOLIC PART | GO:0044445 | pos | 2.191486 | 0.021 |
| TCONS_00345892 | PIGMENT GRANULE | GO:0048770 | pos | 2.062599 | 0.039 |
| TCONS_00345892 | MELANOSOME | GO:0042470 | pos | 2.062599 | 0.039 |
| TCONS_00393325 | DNA PACKAGING COMPLEX | GO:0044815 | pos | 2.322331 | 0.025 |
| TCONS_00443100 | MICROVILLUS | GO:0005902 | neg | -2.04671 | 0.029 |

Supplementary Table S7. GO MF annotations of putative lincRNAs by guilt-by-association method.

| probe.ID | GO.name | GO.ID | pos.or.neg | NES | FWER p-val |
| --- | --- | --- | --- | --- | --- |
| TCONS_00042182 | RAB GUANYL-NUCLEOTIDE EXCHANGE FACTOR ACTIVITY | GO:0017112 | neg | -2.11514 | 0.023 |
| TCONS_00079670 | ELECTRON CARRIER ACTIVITY | GO:0009055 | pos | 2.053659 | 0.027 |
| TCONS_00093223 | DRUG BINDING | GO:0008144 | neg | -2.18521 | 0.015 |
| TCONS_00093223 | ELECTRON CARRIER ACTIVITY | GO:0009055 | neg | -2.11039 | 0.026 |
| TCONS_00093223 | ACTIN MONOMER BINDING | GO:0003785 | neg | -2.07847 | 0.03 |
| TCONS_00119949 | PEPTIDASE REGULATOR ACTIVITY | GO:0061134 | pos | 2.152569 | 0.014 |
| TCONS_00145101 | PYRIDOXAL PHOSPHATE BINDING | GO:0030170 | pos | 2.159046 | 0.013 |
| TCONS_00149399 | MONOCARBOXYLIC ACID BINDING | GO:0033293 | pos | 2.178964 | 0.009 |
| TCONS_00149399 | CHANNEL INHIBITOR ACTIVITY | GO:0016248 | pos | 2.106354 | 0.026 |
| TCONS_00149399 | ION CHANNEL INHIBITOR ACTIVITY | GO:0008200 | pos | 2.087313 | 0.034 |
| TCONS_00169879 | STRUCTURAL MOLECULE ACTIVITY | GO:0005198 | pos | 2.07599 | 0.045 |
| TCONS_00176787 | PEPTIDASE REGULATOR ACTIVITY | GO:0061134 | pos | 2.209805 | 0.011 |
| TCONS_00176787 | STRUCTURAL MOLECULE ACTIVITY | GO:0005198 | pos | 2.175961 | 0.022 |
| TCONS_00176787 | OXIDOREDUCTASE ACTIVITY | GO:0016491 | pos | 2.162677 | 0.024 |
| TCONS_00176787 | PEPTIDASE ACTIVATOR ACTIVITY | GO:0016504 | pos | 2.128619 | 0.039 |
| TCONS_00183900 | CARBON-OXYGEN LYASE ACTIVITY | GO:0016835 | pos | 2.109415 | 0.048 |
| TCONS_00261147 | STRUCTURAL MOLECULE ACTIVITY | GO:0005198 | pos | 2.133871 | 0.018 |
| TCONS_00320879 | PYRIDOXAL PHOSPHATE BINDING | GO:0030170 | pos | 2.087586 | 0.027 |
| TCONS_00354155 | STRUCTURAL MOLECULE ACTIVITY | GO:0005198 | pos | 2.105842 | 0.025 |
| TCONS_00354155 | PEPTIDASE REGULATOR ACTIVITY | GO:0061134 | pos | 2.074478 | 0.043 |
| TCONS_00215988 | PEPTIDASE REGULATOR ACTIVITY | GO:0061134 | pos | 2.107961 | 0.035 |
| TCONS_00302028 | GLYCOSAMINOGLYCAN BINDING | GO:0005539 | neg | -2.1177 | 0.024 |
| TCONS_00387179 | CYSTEINE-TYPE ENDOPEPTIDASE INHIBITOR ACTIVITY | GO:0004869 | pos | 2.094686 | 0.031 |
| TCONS_00397901 | PYRIDOXAL PHOSPHATE BINDING | GO:0030170 | pos | 2.095065 | 0.032 |
| TCONS_00401892 | PYRIDOXAL PHOSPHATE BINDING | GO:0030170 | pos | 2.118087 | 0.023 |
| TCONS_00034801 | STRUCTURAL MOLECULE ACTIVITY | GO:0005198 | pos | 2.294604 | 0.017 |
| TCONS_00034801 | CALCIUM-DEPENDENT PROTEIN BINDING | GO:0048306 | pos | 2.175051 | 0.047 |
| TCONS_00017904 | DRUG BINDING | GO:0008144 | neg | -2.07071 | 0.032 |
| TCONS_00023496 | MITOGEN-ACTIVATED PROTEIN KINASE BINDING | GO:0051019 | pos | 2.125582 | 0.009 |

**REFERENCES**

60 Guttman, M. *et al.* Ab initio reconstruction of cell type-specific transcriptomes in mouse reveals the conserved multi-exonic structure of lincRNAs. *Nature biotechnology* **28**, 503-510, doi:10.1038/nbt.1633 (2010).

61 Jia, J. *et al.* Regulation of pluripotency and self- renewal of ESCs through epigenetic-threshold modulation and mRNA pruning. *Cell* **151**, 576-589, doi:10.1016/j.cell.2012.09.023 (2012).

62 Blackledge, N. P. *et al.* Variant PRC1 complex-dependent H2A ubiquitylation drives PRC2 recruitment and polycomb domain formation. *Cell* **157**, 1445-1459, doi:10.1016/j.cell.2014.05.004 (2014).

63 Carter, A. C., Davis-Dusenbery, B. N., Koszka, K., Ichida, J. K. & Eggan, K. Nanog-independent reprogramming to iPSCs with canonical factors. *Stem cell reports* **2**, 119-126, doi:10.1016/j.stemcr.2013.12.010 (2014).

64 Boo, K. *et al.* Pontin functions as an essential coactivator for Oct4-dependent lincRNA expression in mouse embryonic stem cells. *Nature communications* **6**, 6810, doi:10.1038/ncomms7810 (2015).

65 Geula, S. *et al.* Stem cells. m6A mRNA methylation facilitates resolution of naive pluripotency toward differentiation. *Science* **347**, 1002-1006, doi:10.1126/science.1261417 (2015).

66 Brind'Amour, J. *et al.* An ultra-low-input native ChIP-seq protocol for genome-wide profiling of rare cell populations. *Nature communications* **6**, 6033, doi:10.1038/ncomms7033 (2015).

67 Xue, Z. *et al.* Genetic programs in human and mouse early embryos revealed by single-cell RNA sequencing. *Nature* **500**, 593-597, doi:10.1038/nature12364 (2013).

68 Auclair, G. *et al.* EHMT2 directs DNA methylation for efficient gene silencing in mouse embryos. *Genome research* **26**, 192-202, doi:10.1101/gr.198291.115 (2016).

69 Tena, J. J. *et al.* Comparative epigenomics in distantly related teleost species identifies conserved cis-regulatory nodes active during the vertebrate phylotypic period. *Genome research* **24**, 1075-1085, doi:10.1101/gr.163915.113 (2014).

70 Sauvageau, M. *et al.* Multiple knockout mouse models reveal lincRNAs are required for life and brain development. *eLife* **2**, e01749, doi:10.7554/eLife.01749 (2013).

71 Yue, F. *et al.* A comparative encyclopedia of DNA elements in the mouse genome. *Nature* **515**, 355-364, doi:10.1038/nature13992 (2014).

72 Mikkelsen, T. S. *et al.* Genome-wide maps of chromatin state in pluripotent and lineage-committed cells. *Nature* **448**, 553-560, doi:10.1038/nature06008 (2007).

73 Goren, A. *et al.* Chromatin profiling by directly sequencing small quantities of immunoprecipitated DNA. *Nature methods* **7**, 47-49, doi:10.1038/nmeth.1404 (2010).

74 Teif, V. B. *et al.* Genome-wide nucleosome positioning during embryonic stem cell development. *Nature structural & molecular biology* **19**, 1185-1192, doi:10.1038/nsmb.2419 (2012).

75 Teif, V. B. *et al.* Nucleosome repositioning links DNA (de)methylation and differential CTCF binding during stem cell development. *Genome research* **24**, 1285-1295, doi:10.1101/gr.164418.113 (2014).
